# Differentiation for drought strategy but conserved plasticity to heat and drought in locally adapted populations of *Arabidopsis thaliana*

**DOI:** 10.1093/aob/mcag153

**Published:** 2026-05-27

**Authors:** Sophia F Buysse, Victoria A Nicholes, Jeffrey K Conner, Emily B Josephs

**Affiliations:** Plant Biology Department, Michigan State University, East Lansing, MI 48824, USA; Ecology, Evolution, and Behavior Program, Michigan State University, East Lansing, MI 48824, USA; Kellogg Biological Station, Hickory Corners, MI 49060, USA; Plant Resilience Institute, Michigan State University, East Lansing, MI 48824, USA; Department of Biology, West Virginia University, Morgantown, WV 26505, USA; Plant Biology Department, Michigan State University, East Lansing, MI 48824, USA; Ecology, Evolution, and Behavior Program, Michigan State University, East Lansing, MI 48824, USA; Kellogg Biological Station, Hickory Corners, MI 49060, USA; Plant Biology Department, Michigan State University, East Lansing, MI 48824, USA; Ecology, Evolution, and Behavior Program, Michigan State University, East Lansing, MI 48824, USA; Plant Resilience Institute, Michigan State University, East Lansing, MI 48824, USA

**Keywords:** *Arabidopsis thaliana*, drought, drought avoidance, drought escape, drought strategy, genetic differentiation, heat, local adaptation, phenotypic plasticity

## Abstract

**Background and Aims:**

Adaptive plasticity may be crucial for plants to survive rapid environmental changes long enough for evolutionary adaptation to occur. Both heat and drought are abiotic stresses projected to increase globally, and their individual impact on plant growth and function has been well-characterized. Drought response, in particular, is categorized into three drought survival strategies: escape, avoidance and tolerance. However, to understand potential responses to climate change, we must manipulate multiple stressors simultaneously. We do not know what role plasticity to the combination of heat and drought plays in drought strategy or how plasticity will affect adaptation to projected future climates.

**Methods:**

We measured heat- and drought-related traits as well as fitness in two locally adapted populations of *Arabidopsis thaliana* from Rödåsen, Sweden, and Castelnuovo di Porto, Italy. We used chamber common gardens that simulate the current autumn and spring climate in Sweden and a hotter and drier climate to identify population differentiation for trait means, trait plasticity and fitness.

**Key Results:**

Population differentiation in both treatments suggests that the Swedish population avoids drought by investing in stress-tolerant leaves while the Italian population escapes drought by flowering early. Despite these differences, there is little evidence for genetic differentiation of plasticity; when experiencing heat and drought, both populations shift their traits in the direction expected to avoid drought. Further, both populations have greatly decreased fitness in heat and drought.

**Conclusions:**

These results highlight that locally adapted populations with genetic differentiation for traits within a single environment can respond to the same stressors with plasticity in the same direction. Further, the combination of increased heat and drought will be extremely damaging to plant populations.

## INTRODUCTION

Plants need to respond through plasticity or genetic change to persist during rapid environmental shifts (Franks *et al*., 2014). Plasticity is the ability of a genotype to produce different phenotypes in response to different environmental conditions (Bradshaw, 1965). Adaptive plasticity allows individuals to change their phenotype to move toward the fitness optimum in different environments (Dudley and Schmitt, 1996; Price *et al*., 2003; Ghalambor *et al*., 2007; Bell and Galloway, 2008). Plasticity is expected to evolve in environments with predictable temporal variation and environments with spatial variation that are connected by gene flow (Bradshaw, 1965; Scheiner, 1998; Sultan and Spencer, 2002; Van Buskirk, 2002; Kawecki and Ebert, 2004). Adaptively plastic populations are more likely to survive rapid environmental changes through an immediate plastic response and persist long enough to genetically adapt to new environments (Bradshaw, 1965; Price *et al*., 2003, 2008; Yeh and Price, 2004; Ghalambor *et al*., 2007; Handelsman *et al*., 2013; Morris, 2014; Diamond and Martin, 2021; Pfennig, 2021).

How plants respond to rapid environmental change will depend on how their past evolutionary history has shaped both trait means and trait plasticity. Within-species and within-population genetic variation for trait means in a single environment and for trait plasticity between environments is common and has been frequently documented in plants (Dudley and Schmitt, 1996; Gianoli and González-Teuber, 2005; Bell and Galloway, 2008; Murren *et al*., 2014). Experimentally, plasticity is measured by growing individuals of the same genotype in multiple environments, but in nature, plasticity can occur due to spatial environmental variation across environments connected by gene flow or within a single individual experiencing temporal environmental variation. Exposing plant populations to environments that simulate combinations of abiotic stresses projected to occur in future climates is crucial to predict adaptation and inform conservation (Suzuki *et al*., 2014; Hamann *et al*., 2021; Zandalinas and Mittler, 2022). We are particularly interested in plasticity to heat and drought because both are projected to increase globally in addition to changes in climate variability (IPCC, 2021).

Escape, avoidance and tolerance are well-studied strategies for adapting to drought stress (Ludlow, 1989) but their specific definitions can vary between studies. Plants using the escape strategy show rapid growth to set seed before a drought is lethal; they are found in locations where a drought ends the growing season. Traits associated with escape include early flowering, low water use efficiency (i.e. use more water in biomass production) and minimal investment in protective metabolites and proteins (Ludlow, 1989; Kenney *et al*., 2014). If caught in a drought, escape plants show signs of stress including wilting or decreased relative water content (Blum, 2014). In contrast, avoidant plants experience drought during their life cycle, rather than at the end of the growing season, and maintain their leaf relative water content to avoid wilting (Ludlow, 1989). Traits associated with avoidance include slow growth, later flowering, high water use efficiency and increased root-to-shoot biomass ratio. Avoidant plants invest in stress protective pathways to allow metabolic and stomatal adjustments, which can be reflected in leaves with low specific leaf area (SLA) and high leaf dry matter content (Ludlow, 1989; Des Marais *et al*., 2012; Kenney *et al*., 2014; Kooyers, 2015). Tolerant plants have tissues that can survive periods of severe dehydration and remain functional during severe water reduction, such as resurrection plants (Ludlow, 1989). We follow these descriptions and define escape plants as quickly flowering to finish their life cycle before the onset of drought, avoidant plants as slow growing to maintain high relative water content and tolerant plants as continuing to function at extremely low water content.

Plants may have traits associated with more than one drought strategy (Levitt, 1980; Ludlow, 1989). While drought strategies are typically used to describe differences between species, variation in drought strategy can occur between populations of the same species. For example, *Arabidopsis thaliana* is generally drought avoidant but some populations from across the European range flower early and have lower water use efficiency consistent with drought escape (Mckay *et al*., 2003; Verslues and Juenger, 2011; Lovell *et al*., 2013; Kenney *et al*., 2014). Previous work identified *FRI* as a pleiotropic gene involved in variation between drought escape and avoidance in *A. thaliana* where low expression *FRI* alleles are associated with early flowering, low water use efficiency and faster growth while high expression is associated with later flowering, higher water use efficiency and slow growth (Mckay *et al*., 2003; Juenger *et al*., 2005; Lovell *et al*., 2013).

Understanding a population’s plastic response to future climates requires experimentally manipulating multiple abiotic stressors in tandem, particularly heat and drought (Anderson, 2016; Jiang *et al*., 2025; Secomandi *et al*., 2025). In outcrossing *Arabidopsis lyrata*, the predicted ability to adapt to heat and drought simultaneously was greatly reduced compared to the ability to adapt to drought alone (Heblack *et al*., 2024). Like drought, heat is a well-characterized abiotic stress. In *A. thaliana*, heat decreases growth, increases SLA and decreases stomatal density among many physiological and molecular responses (Vasseur *et al*., 2011; Vile *et al*., 2012; Suter and Widmer, 2013; Stewart *et al*., 2016; Gao *et al*., 2020). Heat can exacerbate the negative effect of drought (Zhang and Sonnewald, 2017; Schepers *et al*., 2024); in *A. thaliana*, the rate of leaf production decreased 16 % in heat and 23 % in drought but 40 % when heat and drought were experienced together (Vile *et al*., 2012). At the molecular level, expression of heat shock proteins, a family of proteins produced in response to heat stress, increased more when heat and drought were experienced together than when either stress was induced alone (Rizhsky *et al*., 2004). However, for traits such as SLA, root-to-shoot ratio and stomatal density, plastic responses to drought and heat are in opposite directions that cancel out when heat and drought are experienced together (Vile *et al*., 2012; Zhang and Sonnewald, 2017). The combination of heat and drought, whether their individual effects are in the same or opposite directions, may determine population persistence in future climates.

We investigated drought strategy, plasticity and fitness in response to combined heat and drought in two locally adapted populations of *A. thaliana* from Rödåsen, Sweden, and Castelnuovo di Porto, Italy (hereafter ‘Sweden’ and ‘Italy’; Ågren and Schemske, 2012). Natural populations of *A. thaliana* are ideal for our study because genetic replicates can be easily grown in chamber common gardens to induce abiotic stress and because *A. thaliana* is 95 % self-fertilizing (Abbott and Gomes, 1989), so seed number encompasses both male and female fitness. Both populations are winter annuals that emerge in the autumn, overwinter as rosettes and flower in the spring (Ågren and Schemske, 2012). The Swedish field site has less precipitation and more variation in water availability than the Italian field site between *A. thaliana* germination and flowering (Mojica *et al*., 2016). The temperature at both sites is similar during germination and flowering, but the Swedish site is much colder during the intermediate months as the soil temperature is below freezing for multiple weeks in the winter (Dittmar *et al*., 2014; Mojica *et al*., 2016; Durán *et al*., 2022). The Italian field site is hotter and drier than the Swedish site in the summer months, typically at the end of flowering, and the Italian population produces seeds with strong dormancy to survive in the seed bank until conditions are favourable (Postma *et al*., 2016).

In both reciprocal transplant studies and chamber common gardens, the Swedish population avoids drought, indicated by higher water use efficiency and later flowering than the Italian population. Adding heat to drought could disrupt or reinforce genetic differentiation for drought strategy between these two populations. The Swedish population is expected to be more plastic to combined heat and drought because the Swedish environment is more variable in temperature and precipitation. This prediction is supported when heat and drought are applied independently (Mojica *et al*., 2016; Stewart *et al*., 2016). Conversely, the Italian population is adapted to a mild winter with fewer calendar days between germination and flowering; it flowers early to escape the severe drought during the hot Italian summer (Ågren and Schemske, 2012; Ågren *et al*., 2013; Dittmar *et al*., 2014; Royer *et al*., 2016; Stewart *et al*., 2016). Because the Swedish and Italian field sites differ in temporal variability of heat and drought, there may have been past selection for differentiation for plasticity to heat and drought. Genetic differentiation for plasticity could result from plasticity in the same direction but of different magnitude or plasticity in opposite directions between these two populations (i.e. selection to delay flowering during drought in the Swedish population results in plasticity for later flowering, and selection to accelerate flowering during drought in the Italian population results in plasticity for earlier flowering).

Much of the prior work on these populations used only one genotype per population (Cohu *et al*., 2013; Stewart *et al*., 2015, 2016; Mojica *et al*., 2016; Durán *et al*., 2022) and/or investigated seed dormancy, cold tolerance, germination timing, flowering time or fitness (Ågren and Schemske, 2012; Ågren *et al*., 2013, 2017; Grillo *et al*., 2013; Dittmar *et al*., 2014; Oakley *et al*., 2014, 2015, 2018, 2023; Postma *et al*., 2016; Postma and Ågren, 2016, 2018; Price *et al*., 2018; Sanderson *et al*., 2020; Zacchello *et al*., 2020; Ellis *et al*., 2021; Ellis and Ågren, 2024; Lee *et al*., 2024). We build on this work by evaluating plasticity in a range of heat- and drought-related traits (days to flowering, relative water content, specific leaf area, stomatal density, and biomass allocation) to a simulated hot and dry future climate and measuring fitness of multiple genotypes per population. If we accurately simulated the native Swedish environment, we expect the Swedish population to have higher fitness in the Current treatment due to local adaptation. However, in the Future treatment, the Italian population may have higher fitness if the increased heat and drought favours drought escape over drought avoidance.

We grew native genotypes from Sweden and Italy in two chamber common garden experiments that each simulated the current autumn and spring climate in Sweden and a hotter and drier potential future Swedish climate. We investigated the following questions:

1. By combining drought with heat to simulate a potential future Swedish climate, do we still observe that Sweden avoids drought and Italy escapes drought?
2. Assuming Sweden avoids drought and Italy escapes drought, is there genetic differentiation for plasticity to a both hot and dry potential future Swedish climate between these two populations that evolved in climates that differ in heat and drought variability?
3. How does a potential future Swedish climate influence differences in fitness between these locally adapted populations?

We found consistent genetic differentiation for drought strategy where Italy escapes and Sweden avoids when heat and drought are experienced together. Despite this, both populations had plasticity in the same direction toward drought avoidance at a similar magnitude for almost all traits. There was no difference in fruit number between the populations within each treatment, perhaps due to our lack of sub-zero temperatures during the simulated winter, but there was local adaptation of Sweden in the current environment as measured by seeds per fruit. Both populations greatly decreased fitness in our simulated future climate with no population differences in either fruit number or seeds per fruit under heat and drought. Despite difficulties simulating Swedish winter in our chamber common gardens, this raises concerns about population persistence as heat and drought stress increase.

## MATERIALS AND METHODS

We conducted two similar growth chamber common garden experiments to investigate plasticity to heat and drought. The first was conducted in 2021 to assess plasticity in a set of heat- and drought-related traits and lifetime fitness. We followed up on this experiment in 2022 with greater genotypic replication in each of the populations to again measure heat- and drought-related leaf traits and focus on biomass allocation rather than lifetime fitness.

### 2021 Experiment

#### Seed material and growing conditions

Seeds from separate maternal plants in Rödåsen, Sweden, and Castelnuovo di Porto, Italy, were collected in the field (Ågren and Schemske, 2012). Native genotypes were grown in chamber common gardens for at least one generation. These native genotypes included the parents of a set of recombinant inbred lines (Ågren *et al*., 2013), but no recombinant inbred lines were grown in this study. We sowed seeds (3–5 per pot) from eight Italian genotypes and 13 Swedish genotypes directly on wet soil and stratified them at 4 °C for 5 d in the dark. Due to mortality, only six Italian genotypes and 12 Swedish genotypes were included in the analysis. This sample is our maximum possible genotypic representation of the Italian population while we included additional lines from the Swedish population because our treatment simulates the Swedish field site. We focus on population means in this study; because *A. thaliana* is highly self-fertilizing (Abbott and Gomes, 1989) this sample should be representative.

After stratification, pots were split into two common gardens in growth chambers (BioChambers) at Michigan State University, East Lansing, MI, USA. One common garden simulated ‘Current’ climate conditions near the Sweden field collection site in the autumn and spring and the other simulated a ‘Future’ hotter and drier climate at the same location. Each treatment included six replicates of the recombinant inbred line (RIL) parents and two replicates of each of the remaining seven genotypes from Italy and 12 genotypes from Sweden [(6 replicates × 2 RIL parents) + (2 replicates × 7 Italy lines) + (2 replicates × 12 Sweden lines) × 2 treatments = 100 plants]. We included greater replication of the RIL parents because most studies on these two populations use only the RIL parents as genotypes representative of the Swedish or Italian population (Ågren and Schemske, 2012; Ågren *et al*., 2013, 2017; Cohu *et al*., 2013; Grillo *et al*., 2013; Dittmar *et al*., 2014; Oakley *et al*., 2014, 2015, 2018, 2023; Stewart *et al*., 2015, 2016; Mojica *et al*., 2016; Postma *et al*., 2016; Postma and Ågren, 2016, 2018; Price *et al*., 2018; Sanderson *et al*., 2020; Zacchello *et al*., 2020; Ellis *et al*., 2021; Durán *et al*., 2022; Ellis and Ågren, 2024; Lee *et al*., 2024). Plants were grown in a soil mix of two parts Michigan Grower Products SureMix (Michigan Grower Products, Galesburg, MI, USA) to one part Sunshine Redi-Earth (Sun Gro HorticultureAgawam, MA, USA) supplemented with Osmocote 15-9-12 slow-release fertilizer (1 tablespoon fertilizer was split evenly between 25 pots; The Scotts Company, Marysville, OH, USA).

Our treatments simulate the ‘Current’ and ‘Future’ climate at the Swedish field site. Briefly, our temperatures change throughout the growing season to simulate the native Swedish environment and the Future treatment is 4 °C hotter and always drier than the Current treatment. Temperatures in each treatment were determined using data from Weather Underground (weatherunderground.com) spanning 1998–2020. Median day and night air temperatures from the Sundsvall-Timra Airport (49 km from the Sweden field collection site) were used to determine the ‘Current’ treatment conditions. In the field, plants emerge in August and September (Ågren and Schemske, 2012). Accordingly, we split temperatures from 1 August to 31 October into four chronological subsets and used the median day and night temperature for each subset as the chamber temperatures for 1 week each (Supplementary Data Table S1). Daylength was determined by following the same procedure. By the end of October, the mean temperature at the Sweden field site is below 10 °C which is the minimum for our chambers. At this point, plants were thinned to one per pot and vernalized for 5 weeks in a cold room where temperatures varied from 7 to 10 °C during the day and were 8 °C at night. Our low temperatures during the simulated winter were sufficient to induce flowering in both populations though they are much higher than temperatures at the Sweden field site during winter (Dittmar *et al*., 2014; Mojica *et al*., 2016; Zacchello *et al*., 2020; Lee *et al*., 2024). The daylength for each week of our simulated winter was set by splitting daylengths from November through April into five subsets and using the median from each subset.

Plants were then returned to growth chambers to simulate spring. At the Sweden field site, plants flower in May and June (Ågren and Schemske, 2012). We simulated spring using the same approach of the median day temperature, night temperature and daylength but time was not condensed. In the spring simulation, 1 week in the experiment corresponded to 1 week of temperatures from the field to allow plants time to flower. Flowering began while we were simulating June, which is near the end of flowering in the field (Ågren and Schemske, 2012). Through our experimental approach, we condensed approximately 48 weeks in the field (August to June) into 25 weeks in chambers (Supplementary Data Table S1).

The ‘Future’ treatment was 4 °C hotter than the ‘Current’ treatment when simulating autumn and spring (collectively 20 of the 25 weeks) following IPCC projections for Sweden within the next 50 years if global temperatures rise by 2 °C (IPCC, 2021). Winter temperatures were the same between both treatments because our minimum temperature in the Current treatment is already much higher than the average winter temperatures in the field. While our ‘Current’ treatment does not accurately simulate the current winter climate at the Swedish field site, we use the Current and Future nomenclature to encompass the general trend of a hotter and drier climate that we simulate in the autumn and spring.

To manipulate water availability, we added 50 g of our soil mix to each pot in the experiment and to ten additional pots. The additional pots were watered to saturation to determine water holding capacity (100 % gravimetric water content) and oven dried at 60 °C for 1 week to determine dry mass (0 % gravimetric water content). We used the average 0 and 100 % masses from the test pots to calculate the weight required to maintain pots with plants in our treatments at their target gravimetric water content. All pots were well watered when seeds were sowed. The ‘Current’ treatment was then kept at 45 % gravimetric water content throughout the experiment to maintain a well-watered environment (Vile *et al*., 2012). Plants in the ‘Future’ treatment were dried down to 25 % gravimetric water content before vernalization (Vile *et al*., 2012). We sought to maintain a non-lethal drought stress. Because plants showed no visible signs of water stress by the end of vernalization, they were dried down to 10 % gravimetric water content for 3 weeks and further dried to 7.5 % gravimetric water content for the final 12 weeks of the experiment. We maintained an overall difference in soil moisture between treatments even when there was no difference in temperature during the winter simulation. Soil moisture was maintained by daily weighing and watering to account for differences in water use by each plant (Granier *et al*., 2006). Plants were rotated within each chamber once a week to reduce microclimate differences.

#### Phenotyping

All traits except days to emergence were measured while plants were experiencing differences in heat and drought between treatments. We recorded emergence and flowering as phenology timepoints defined as the first day green growth was visible and the first day that a white petal was visible from a bud, respectively, after sowing. On the first day of flowering, the last fully developed rosette leaf was collected to measure leaf traits on a leaf that developed while there were differences in both heat and drought between the treatments. This leaf was typically from the third whorl of leaves. We measured relative water content, SLA and leaf dry matter content as related measures of the leaf economics spectrum (Pérez-Harguindeguy *et al*., 2013). The leaf was weighed immediately after collection (fresh mass: *M*_f_) and scanned to measure leaf area (*A)* with ImageJ (Schneider *et al*., 2012). Each leaf was then rehydrated by wrapping in a wet paper towel and tin foil and placed in the dark at 4 °C for 24–48 h. Rehydrated leaves were weighed (*M*_r_) and dried at 45 °C for at least 2 weeks before recording dry mass (*M*_d_). From these values, we calculated relative water content ((*M*_f_ − *M*_d_)/(*M*_r_ − *M*_d_)), SLA (*A*/*M*_d_) and leaf dry matter content (*M*_d_/*M*_r_). Relative water content on the first day of flowering is our measurement of physiological drought stress where a decrease in relative water content in the Future treatment indicates successful drought stress (VanBuren *et al*., 2024). We measured relative water content at the same developmental stage in both treatments on adult leaves that were not senescing.

Plants were destructively harvested in the final 2 weeks of the experiment when they fit any of the following criteria: (1) three fruits dehisced on the main flowering stalk and few open flowers or buds remain; (2) two fruits dehisced on the main flowering stalk and most fruits on branches dehisced; (3) no remaining open flowers or green buds, even if no fruits dehisced; (4) 175 d post-planting (25 weeks; simulating the end of July in the field) if they had not met the earlier criteria. Only five of the 81 plants that survived were harvested following the final criterion; all were Sweden genotypes and four were in the Current treatment. At harvest, we counted rosette leaf number, and dried rosette and reproductive biomass at 45 °C for at least 2 weeks to analyse reproductive allocation. We manually counted fruit number in ImageJ with photos of all plant branches and collected ten fruits from the main flowering stalk of each plant to calculate the average seed weight and the average number of seeds per fruit. To measure total fitness, the total number of fruits produced by a plant was multiplied by the average number of seeds per fruit. This is an unusually complete measure of total male and female fitness because *A. thaliana* is 95 % self-fertilizing (Abbott and Gomes, 1989; Platt *et al*., 2010).

Plasticity in response to the Future treatment was measured by subtracting the mean trait value for a given genotype in the Current treatment from that genotype’s mean trait value in the Future treatment.

#### Statistical analysis

All statistical analyses were done in R (v.4.4.1; R Core Team, 2024) with the following packages: *tidyverse*, *lme4*, *lmerTest* and *emmeans* (Bates *et al*., 2015; Kuznetsova *et al*., 2017; Wickham *et al*., 2019; Lenth, 2024). The Current and Future treatments were analysed using type III ANOVAs (Des Marais *et al*., 2013; Laitinen and Nikoloski, 2019) with treatment, population and their interaction as fixed effects and genotype nested within population as a random effect. The treatment fixed effect tests for plasticity across both populations, the population fixed effect tests for population differentiation for trait means across the treatments, and their interaction tests for population differentiation for plasticity. SLA and average seed number per fruit were log transformed to increase the normality of model residuals. Model predicted means and 95 % confidence intervals were plotted with *ggplot* (Wickham, 2016); log-transformed traits were back transformed for plotting. We interpret genetic differentiation and plasticity in terms of drought escape or drought avoidance. These terms describe suites of trait means that occur together according to drought strategy definitions.

### 2022 Experiment

#### Seed material and growing conditions

We conducted a follow-up experiment in April 2022 using similar methods but focusing on allocation to above- and below-ground biomass at bolting instead of lifetime fitness. Root-to-shoot biomass ratio increases under severe drought to access additional soil water and reduce leaf transpiration (Verslues and Juenger, 2011). Eight pots were directly sown with seeds from each of the eight Italian genotypes and eight randomly chosen Swedish genotypes that were grown in the 2021 experiment. Four replicates of each genotype were planted in each treatment (16 genotypes × 4 replicates × 2 treatments = 128 plants). Plants were grown in Sunshine Redi-Earth amended with 3.6 kg m^−3^ 15-9-12 Osmocote slow-release fertilizer. Soil mass, water holding capacity and dried mass were determined as in the 2021 experiment. Seeds were stratified in the dark at 8 °C for 5 d before being moved to a BioChambers growth chamber at Michigan State University. Due to time constraints, all pots were grown in 16 °C day temperatures, 13 °C night temperatures with 16-h days for 3 weeks (Supplementary Data Table S2). Plants were then thinned to one per pot or transplanted to new pots to replace low survival of certain genotypes (Italy only). After thinning and transplanting, each population had the same number of plants, but some Italian genotypes had more than four replicates per treatment and some had fewer (min = 1, max = 6; Current treatment mean = 4.57, Future treatment mean = 4.43). Due to mortality, only seven of the Italian genotypes were included in the final analysis.

All pots were vernalized as in the 2021 experiment (Supplementary Data Table S2). After vernalization, plants were moved to Percival Growth Chambers with photosynthetically active radiation of 88–90 µmol m^−2^ s^−1^ at the Kellogg Biological Station, Hickory Corners, MI, USA. Plants were randomized within two blocks for each treatment, and each block was grown in a different growth chamber. Chambers simulated Current and Future (+4 °C) spring using the same temperatures and daylengths from the 2021 experiment (Table S2; heat and drought treatment for 10 of the 18 weeks). In the 2021 experiment, we noticed that light levels were lower in the Current treatment which had lower temperatures (during week 13 of the 2021 experiment, the Current chamber had an average of 1551.833 lux at 10 °C while the Future chamber had an average of 2250.611 lux while at 14 °C). To remedy this, in 2022 shelf heights within the chamber were adjusted each week as temperatures were changed to ensure a consistent 88–90 µmol m^−2^ s^−1^ of light at the soil surface and plants were rotated weekly to reduce microclimate differences. We focus our conclusions on results shared between both experiments that are probable from heat and drought rather than results that only occurred in 2021 potentially from differences in light. However, not all traits were measured in both years. Days to flowering and fitness were only measured in 2021 while biomass allocation and stomatal density were only measured in 2022 (see *Phenotyping*).

As in 2021, the Current treatment was consistently maintained at 45 % soil moisture. The Future treatment began at 7.5 % soil moisture, which was increased to 10 % in week 14 due to observed wilting and drying of rosette leaves. Plants were weighed and watered daily during the first 3 weeks of the experiment, twice a week during vernalization and daily during the 8 weeks of spring simulation.

#### Phenotyping

Emergence, rosette leaf number, SLA, leaf dry matter content and relative water content were measured as in the 2021 experiment with the following exceptions: leaf traits were measured at bolting, the day buds were visible in the centre of the rosette, rather than at flowering and biomass was dried at 60 °C for at least 48 h before weighing. A leaf in the third or fourth whorl from the apical meristem was selected for a stomatal peel on the abaxial side of the leaf to measure stomatal density at bolting. Stomata density may be locally adapted in *A. thaliana* though it is not correlated with water use efficiency (Dittberner *et al*., 2018; de Ollas *et al*., 2023). The whorl selected depended upon the size of the plant as small leaves do not peel cleanly. A thin layer of New-Skin Liquid Bandage (Advantice Health, Cedar Knolls, NJ, USA) was painted onto the abaxial side of the selected leaf and allowed to dry. Using clear packing tape, the stomata peel (∼1 cm^2^) was carefully removed from the leaf and transferred onto a glass microscope slide for imaging. Two images were taken at 400× magnification for each stomatal peel; each field of view was 0.06612 mm^2^. Images were taken at randomly chosen spots on the peels but avoiding overlapping windows. Only stomata entirely in the field of view of the image were counted in ImageJ to determine stomatal density.

Plants were destructively harvested at bolting, or as soon as possible after, to obtain root and shoot biomass (range 0–10 d after bolting; median = 4 d). Shoot biomass was collected by cutting plants at the soil surface. Each pot was then placed in a 1 % Liquinox solution to soak for 20 min. Soil was gently washed away with running water until roots were clean or 30 min had passed; most roots were clean within the 30-min timeframe. Root and shoot biomass was dried at 60 °C for 3 d and weighed.

#### Statistical analysis

All analyses were conducted as in the 2021 experiment with the addition of block nested in treatment as a fixed effect in the model. Stomatal density, SLA, shoot biomass and root-to-shoot ratio were log transformed to increase normality of residuals before analysis. Model output was back-transformed before plotting for comparison across traits and studies.

## RESULTS

**Fig. 1.**
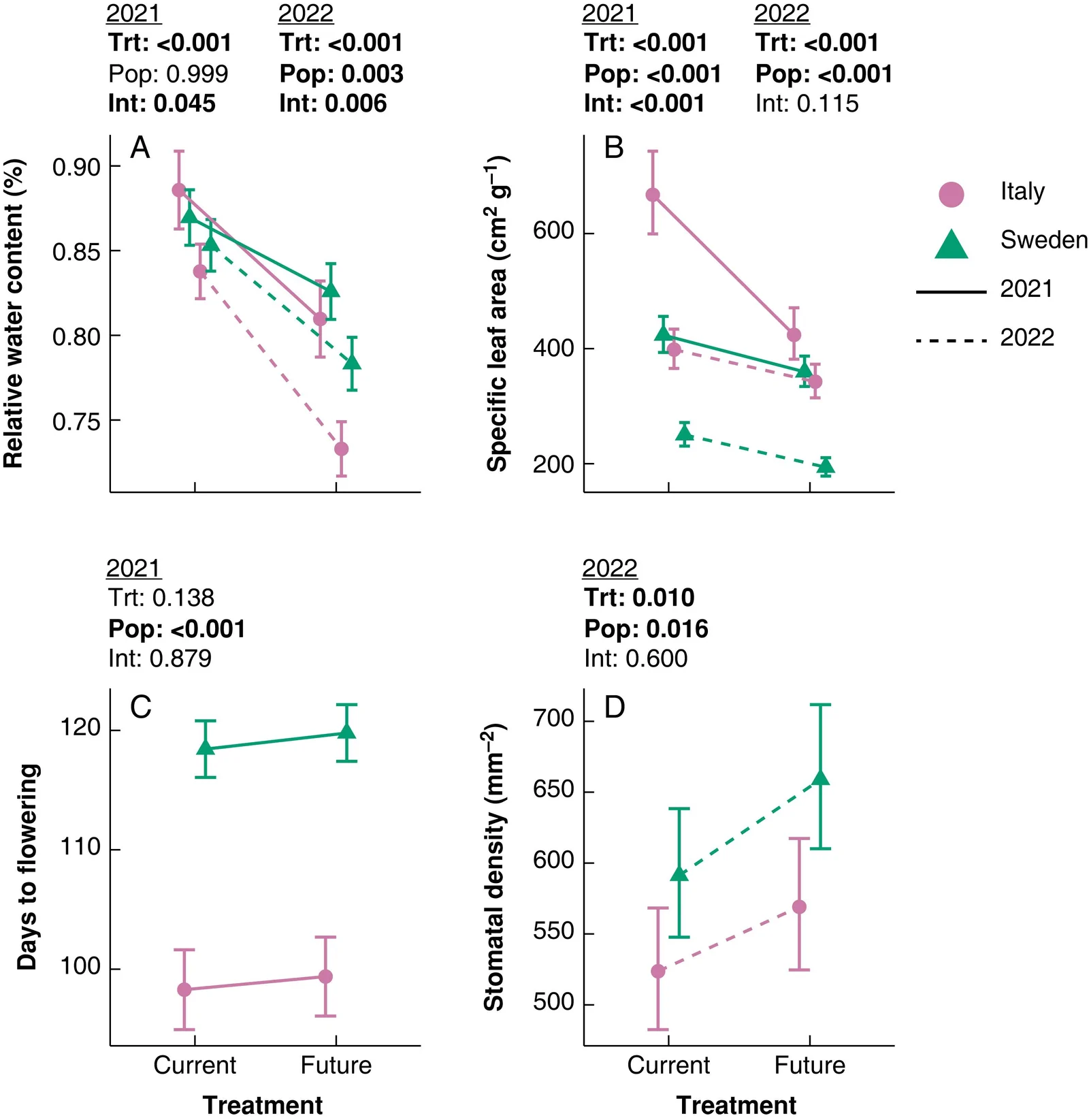
Sweden avoids and Italy escapes combined heat and drought. Each panel displays estimated marginal means for Sweden (triangles) and Italy (circles) with 95 % confidence intervals; the Future treatment is both hotter and drier. Lines connecting means illustrate plasticity; the 2021 experiment is connected with solid lines and the 2022 experiment with dotted lines. Statistics above each panel show *P*-values for ANOVA fixed effects.

Our heat and drought treatment successfully induced non-lethal water stress as leaf relative water content decreased at bolting (2022) and flowering (2021). Relative water content decreased in both populations in the Future treatment in both experiments, and more so in 2022; population mean relative water content decreased by 8 % (Italian population, 2021), 10 % (Italian population, 2022), 4 % (Swedish population, 2021) and 7 % (Swedish population, 2022) (Fig. 1A; 2021 Treatment *P* = 1.93 × 10^−10^; 2022 Treatment *P* = 1.18 × 10^−14^).

### Sweden avoids, Italy escapes

**Fig. 2.**
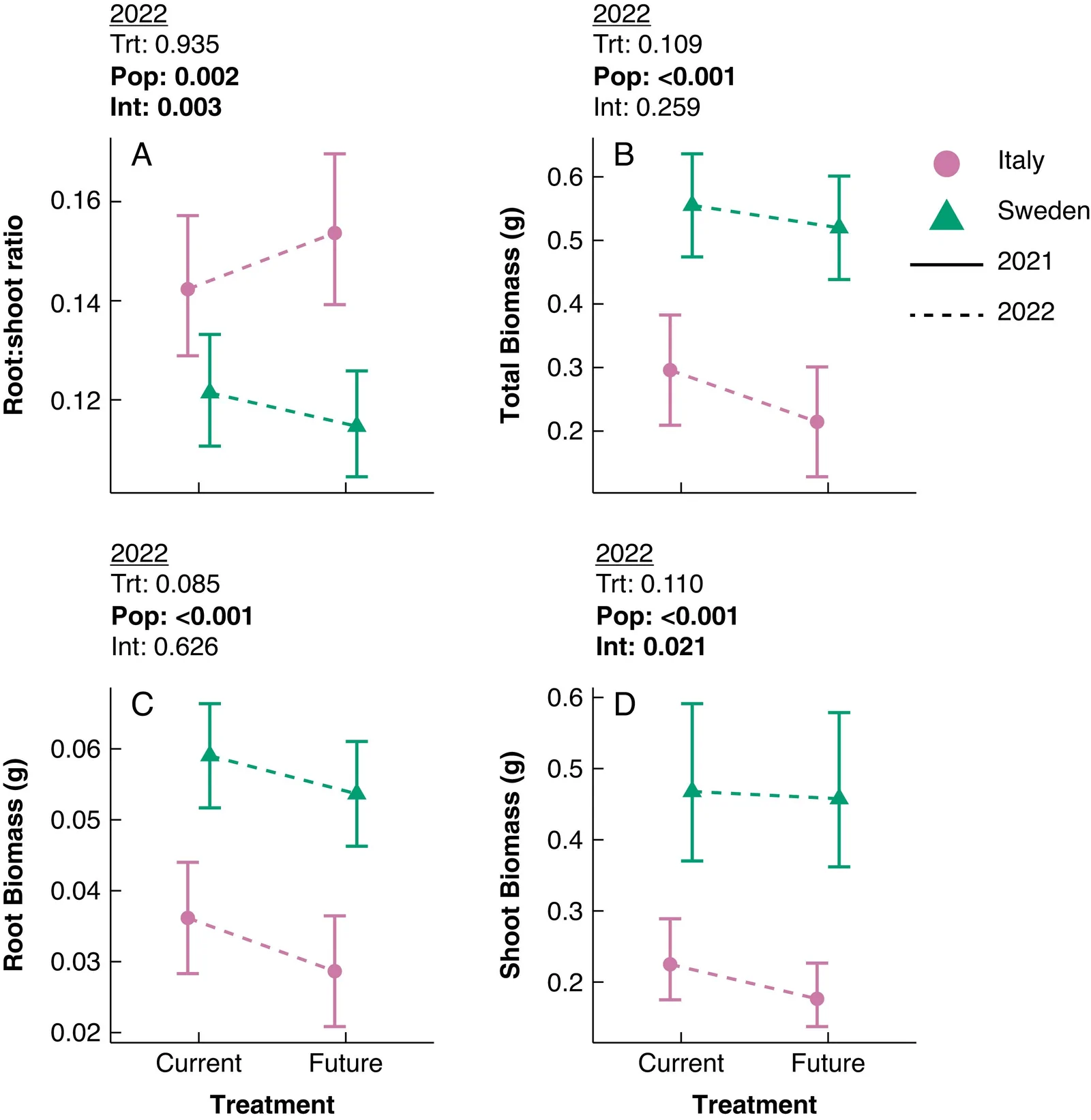
Population genetic differentiation for plasticity in root-to-shoot biomass ratio is driven by a greater decrease in shoot biomass than root biomass in Italy. Each panel displays estimated marginal means for Sweden (triangles) and Italy (circles) with 95 % confidence intervals; the Future treatment is both hotter and drier. Lines connecting means illustrate plasticity; these traits were only measured in 2022 (dotted lines). Statistics above each panel show *P*-values for ANOVA fixed effects.

Consistent with genetic differentiation for drought avoidance, the Swedish population had higher relative water content than the Italian population in the Future treatment though the difference was only significant in 2022 (Fig. 1A; 2021 Pop *P* = 0.99; 2022 Pop *P* = 0.003). The Swedish population had 15 % (2021 Future) to 42 % (2022 Current) lower SLA (Fig. 1B; 2021 Pop *P* = 9.11 × 10^−5^; 2022 Pop *P* = 5.38 × 10^−8^), 3 % (2021 Future) to 30 % (2022 Future) higher leaf dry matter content (Supplementary Data Fig. S1A; 2021 Pop *P* = 0.086; 2022 Pop *P* = 8.21 × 10^−10^) and flowered 20 d later (both treatments; Fig. 1C; 2021 Pop *P* = 9.20 × 10^−9^) than Italy. SLA and leaf dry matter content were highly negatively correlated across all plants because *M_d_* is the denominator of SLA and numerator of leaf dry matter content (2021 *r* = −0.687, *P* < 0.001; 2022 *r* = −0.747, *P* < 0.001). Sweden also had 13 % (Current) to 16 % (Future) greater stomatal density than Italy (Fig. 1D; 2022 Pop *P* = 0.016). This suite of trait means in the Swedish population in both treatments is consistent with drought avoidance. Genetic differentiation for root-to-shoot ratio, however, was not consistent with this pattern. The Italian population had a greater root-to-shoot ratio than the Swedish population (Fig. 2A; 2022 Pop *P* = 0.002). Greater root-to-shoot ratio is consistent with drought avoidance rather than escape (Verslues and Juenger, 2011).

### Plasticity is usually in the same direction in both populations

Despite genetic differentiation for traits within each treatment, there was limited genetic differentiation for plasticity: the interaction between genotype and environment was only significant for relative water content (both years), SLA (2021), root-to-shoot ratio (2022) and shoot biomass (2022) (Table 1). The genetic differentiation for plasticity in relative water content, SLA and shoot biomass was driven by plasticity of different magnitudes in the direction expected of drought avoidance: for example, SLA decreased in the Future treatment (Fig. 1B; 2021 Trt *P* = 4.62 × 10^−12^; 2022 Trt *P* = 3.21 × 10^−9^). Root-to-shoot biomass was the only trait with plasticity in opposite directions between populations. There was additional evidence for differentiation for plasticity in the components of relative water content, SLA and leaf dry matter content but neither population was consistently more plastic (Supplementary Data Fig. S1; Table S3). Thus, genetic differentiation for drought strategies seen in trait means did not translate to genetic differentiation for plasticity to heat and drought in either year.

**Table 1.** ANOVA results.

Experiment		2021				2022			
Trait	Term	SumSq	DenDF	*F*	*P*	SumSq	DenDF	*F*	*P*
Days to flowering	Treatment	27.902	61.045	2.261	0.138	Not measured			
	Population	1578.550	15.070	127.925	9.196E−09 ***				
	Interaction	0.290	61.045	0.024	0.879				
Relative water content	Treatment	0.067	63.528	57.293	1.932E−10 ***	0.096	106.910	80.192	1.182E−14 ***
	Population	2.127E−10	14.249	1.812E−07	0.999	0.016	12.603	13.665	0.003 **
	Interaction	0.005	63.528	4.192	0.045 *	0.009	107.134	7.907	0.006 **
	Block	Not applicable				0.003	107.291	1.434	0.243
Stomatal density	Treatment	Not measured				0.023	107.213	6.847	0.010 **
	Population					0.026	12.631	7.793	0.016 *
	Interaction					0.001	107.366	0.276	0.600
	Block					0.002	107.430	0.327	0.722
SLA (log)	Treatment	0.315	59.100	74.537	4.623E−12 ***	0.251	108.472	41.593	3.214E−09 ***
	Population	0.156	10.698	36.814	9.111E−05 ***	0.716	13.283	118.739	5.347E−08 ***
	Interaction	0.070	59.100	16.529	1.436E−04 ***	0.015	108.605	2.531	0.115
	Block	Not applicable				0.050	108.741	4.113	0.019 *
Root-to-shoot (log)	Treatment	Not measured				1.954E−05	107.654	0.007	0.935
	Population					0.041	13.410	14.048	0.002 **
	Interaction					0.026	107.767	9.145	0.003 **
	Block					0.007	107.797	1.272	0.285
Shoot biomass (log)	Treatment	Not measured				0.034	107.305	2.594	0.110
	Population					0.389	13.147	29.844	1.045E−04 ***
	Interaction					0.072	107.397	5.505	0.021 *
	Block					0.004	107.417	0.144	0.866
Root biomass	Treatment	Not measured				4.516E−04	107.981	3.018	0.085 †
	Population					0.004	13.518	26.590	1.629E−04 ***
	Interaction					3.579E−05	108.121	0.239	0.626
	Block					2.388E−04	108.173	0.798	0.453
Total biomass	Treatment	Not measured				0.034	107.504	2.615	0.109
	Population					0.374	13.211	28.991	1.177E−04
	Interaction					0.017	107.626	1.288	0.259
	Block					0.007	107.660	0.279	0.757
Fruit number	Treatment	6.177E+06	71.888	167.345	1.899E−20 ***	Not measured			
	Population	5.016E+04	17.041	1.359	0.260				
	Interaction	5310.171	71.888	0.144	0.706				
Seeds per fruit (log)	Treatment	0.162	69	37.439	5.041E−08 ***	Not measured			
	Population	0.108	69	25.047	4.099E−06 ***				
	Interaction	0.074	69	17.088	9.884E−05 ***				
Total fitness	Treatment	2.183E+10	69.360	150.637	4.720E−19 ***	Not measured			
	Population	1.155E+07	16.507	0.080	0.781				
	Interaction	3.665E+08	69.360	2.529	0.116				

Columns denote the trait used as the response variable in the model, the predictor model terms, and then for the 2021 and 2022 experiments the sum of squares (SumSq), the denominator degrees of freedom (DenDF), *F*-value and *P-*value. Numerator degrees of freedom is always 1 for treatment, population and their interaction; it is always 2 for block. Symbols illustrate *P-*values: ^†^*P* < 0.1; *P < 0.05; **P < 0.01; ***P < 0.001.

There was variation for plasticity within each population but plasticity was typically in the same direction within each population and only varied in magnitude (Fig. S2).

While escape and avoidance are useful terms to understand plant survival to water stress generally, traits associated with each strategy are not prescriptive of all plant traits or trait plasticity. For example, the Italian population had a greater root-to-shoot ratio than the Swedish population (Pop *P* = 0.002) and increased root-to-shoot ratio in the Future treatment. This is typical of drought avoidance rather than escape (Verslues and Juenger, 2011). In contrast, the Swedish population decreased root-to-shoot ratio in the Future treatment (Fig. 2A). The different directions of plasticity in root-to-shoot ratio between the populations were caused by different patterns of plasticity in the component biomasses. Both populations had lower root biomass in the Future treatment (Fig. 2C). The Swedish population maintained consistent shoot biomass across treatments, (Fig. 2D) resulting in a decrease in root-to-shoot ratio. In contrast, the Italian population decreased shoot biomass in the Future treatment (Fig. 2D). Since the values for shoot biomass were an order of magnitude higher than root biomass, this caused an increase in root-to-shoot ratio despite an overall decrease in total biomass (Fig. 2B). The lower root and shoot biomass of the Italian plants and their plastic decrease in the Future treatment is consistent with drought escape by growing quickly and not investing in stress avoidance despite the observed increase in root-to-shoot biomass that is consistent with drought avoidance.

The Roda-11 Swedish genotype was an outlier in all biomass traits collected in 2022 (Supplementary Data Fig. S2). However, this outlier is not driving genetic differentiation between the populations; removing Roda-11 from our analyses increased the plasticity of root biomass and total biomass and strengthened the genetic differentiation for plasticity in root-to-shoot biomass ratio (Table S4).

### Fitness is greatly reduced in the future treatment

**Fig. 3.**
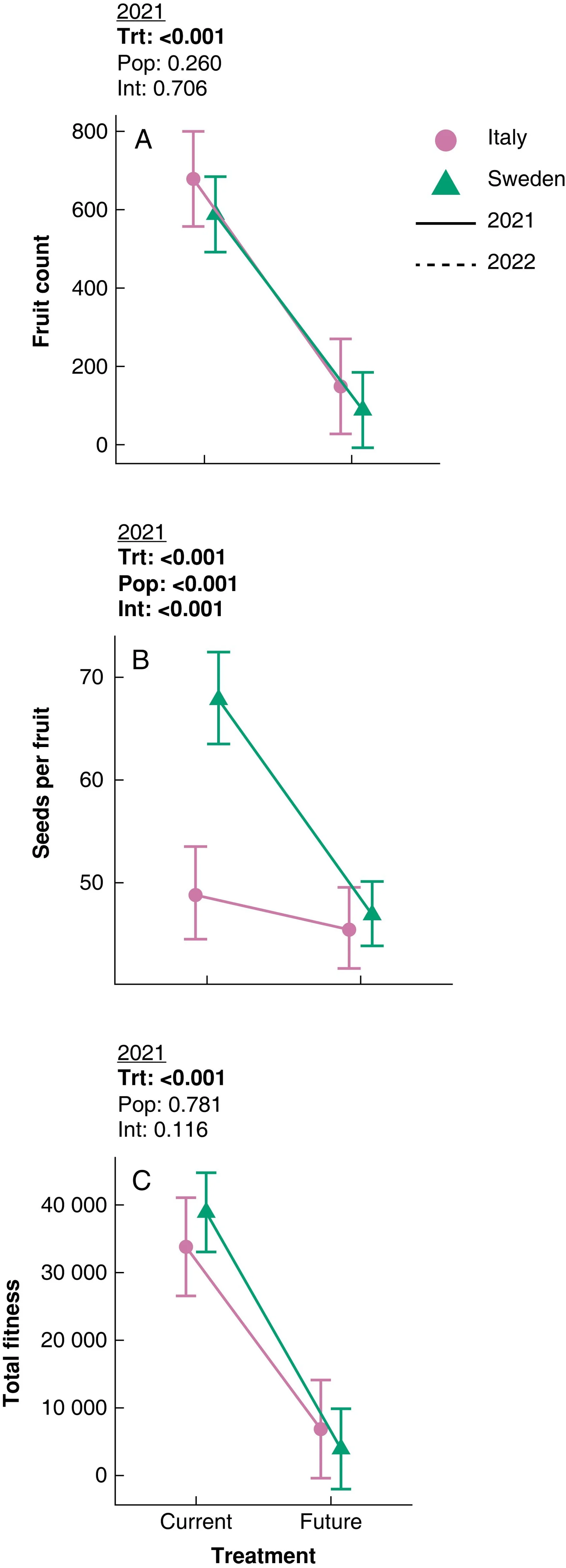
Combined heat and drought greatly decrease fitness for both populations. Each panel displays estimated marginal means for Sweden (triangles) and Italy (circles) with 95 % confidence intervals; the Future environment is both hotter and drier. Lines connecting means illustrate plasticity; fitness was only measured in 2021 (solid lines). Statistics above each panel show *P*-values for ANOVA fixed effects. In fruit count (A) and total fitness (C), plants that produced no fruits are included (Sweden *N* = 30; Italy *N* = 20) but seeds per fruit (B) only includes plants that produced at least one fruit (Sweden *N* = 26; Italy *N =* 16).

Fruit number (Trt *P* = 1.90 × 10^−20^) and total fitness (the average number of seeds per fruit multiplied by fruit number; Trt *P* = 4.72 × 10^−19^) were greatly reduced in both populations in the Future treatment. Fruit number decreased by 78 % in the Italian population and 85 % in the Swedish population. Total fitness decreased by 80 % in the Italian population and 90 % in the Swedish population. However, the average number of seeds per fruit decreased significantly only for the Swedish population (Fig. 3; Trt *P* = 5.04 × 10^−8^; decreased 31 and 7 % in the Swedish and Italian populations, respectively). Average seed weight also decreased by 5 % in the Swedish population and 2 % in the Italian population but the differences were not significant (Supplementary Data Fig. S3).

Overall, there was no differentiation between populations in total fitness in either treatment (Pop *P* = 0.781) but the Future treatment reduced fitness in the Swedish population marginally more than in the Italian population (Int *P* = 0.116) due to the decreased number of seeds per fruit. Despite being smaller plants overall, the Italian plants were able to match the fitness output of the Swedish plants by allocating almost twice as much biomass to reproduction in the Future treatment than Swedish plants (Supplementary Data Fig. S3; Pop *P* = 0.030).

## DISCUSSION

### Adding heat to drought does not disrupt differentiation for drought strategy

Through replicated common garden experiments, we confirmed that *A. thaliana* from Sweden and Italy are genetically differentiated to avoid and escape drought, respectively, even if drought and heat are experienced concurrently. The Swedish population evolved in a climate with periodic drought during the plant life cycle; our results indicate it avoids drought through delayed flowering, greater biomass investment and maintenance of higher relative leaf water content (i.e. not wilting) when water availability is low (Figs 1 and 2). In contrast, the Italian population is adapted to an environment with a terminal drought; it escapes drought by flowering early and investing less in stress tolerant leaves (Figs 1 and 2). Root-to-shoot ratio is an exception to this pattern where the Italian population had an increased root-to-shoot ratio as expected of drought avoidance, but genetic differentiation for total biomass shows that the Italian population is much smaller, consistent with less vegetative growth to escape drought (Fig. 2). Our results use additional genotypes per population grown in heat and drought to confirm drought strategies identified in prior studies of *A. thaliana* with flowering time and water use efficiency in both the field and growth chambers (Ågren and Schemske, 2012; Grillo *et al*., 2013; Dittmar *et al*., 2014; Mojica *et al*., 2016; Ågren *et al*., 2017; Oakley *et al*., 2023). Additionally, our results fit more broadly within a latitudinal gradient in resource-use strategy among European *A. thaliana.* Populations from southern latitudes, like Italy, have a short lifespan with high SLA and northern populations, like Sweden, have a long lifespan and low SLA (Estarague *et al*., 2022).

Our results support prior work that the Swedish population avoids drought, but the absence of cold in both of our treatments complicates our conclusions. First, the same pathways involved in drought avoidance can also be involved in cold tolerance as both increase cellular protective compounds to avoid dehydration (Kong and Henry, 2016; Suprasanna *et al*., 2016). Genotypes of *A. thaliana* from northern Europe had higher survival in both a cold treatment and a hot and dry treatment (Estarague *et al*., 2022). Our simulated winter stayed well above freezing, but cold tolerance is an important aspect of local adaptation of these populations (Oakley *et al*., 2018, 2023; Lee *et al*., 2024). We interpret lower SLA and greater shoot biomass as indicating greater vegetative investment by Sweden in drought avoidant leaves that are less prone to water loss. Those same trait means can also indicate cold tolerance so we cannot separate past selection in the native Swedish environment on drought avoidance from congruent selection on cold tolerance. Second, flowering in *A. thaliana* is controlled by a vernalization requirement in some populations (Michaels and Amasino, 2000). While both the Swedish and Italian populations are ecological winter annuals, the Italian population does not require vernalization to flower while the Swedish population does (Grillo *et al*., 2013). The difference in flowering time we observed between the populations is more similar to differences in flowering time observed in the field at the Italian site where temperatures are above freezing rather than the Swedish site where temperatures are below freezing for multiple weeks and there is little difference in days to flowering between the two populations (Ågren *et al*., 2017). Thus, our observed difference in flowering time may represent genetic differentiation for a vernalization requirement prior to flowering rather than represent genetic differentiation for drought strategy. However, the earlier flowering of the Italian genotypes in both of our treatments is consistent with field experiments and a prior chamber study that did include temperatures below freezing to simulate a Swedish winter. Both studies identified that Italian alleles at flowering time quantitative trait loci cause earlier flowering (Dittmar *et al*., 2014; Ågren *et al*., 2017).

### Locally adapted populations respond to heat and drought stress with similar plasticity

We expected the Swedish population would be more plastic than the Italian population for most traits because Sweden has a more variable environment during the plant life cycle (Mojica *et al*., 2016) and plant populations from more variable precipitation regimes are more plastic for drought response traits (Gianoli and González-Teuber, 2005). This was supported in previous work where the Swedish population was more plastic in response to drought (Mojica *et al*., 2016) and heat (Stewart *et al*., 2016). Our results do not support this prediction. Only a few traits show genetic differentiation for plasticity and neither population was consistently more plastic across traits.

Flowering time is an important indicator of drought strategy where earlier flowering is associated with drought escape and later flowering with drought avoidance. We thus predicted that plasticity of flowering time may be in opposite directions; the drought escaping population may have plasticity for earlier flowering and the drought avoiding population may have plasticity for later flowering. Our results, instead, show both populations have slight plasticity for later flowering, expected of drought avoidance. In fact, plasticity of both populations was in the direction expected of drought avoidance (i.e. generally in the direction of or more extreme than the Sweden mean phenotype in the Current treatment), for all traits except root-to-shoot ratio. While this is contrary to our prediction that populations using different drought strategies may have plasticity in opposite directions for traits such as flowering time or SLA, plasticity in the avoidant direction is not wholly unexpected because avoidance is favoured when plants experience moderate water limitation during their life cycle and *A. thaliana* is generally considered avoidant (Verslues and Juenger, 2011). Thus, drought strategy may contribute to the local adaptation of these populations, but both populations respond to the environmental cues of heat and drought with similar plasticity. This conserved plasticity is in agreement with prior work showing plasticity to mild drought stress is consistent in direction across *A. thaliana* genotypes though there is variation in magnitude (Clauw *et al*., 2015) and a meta-analysis of 34 plant species that showed local adaptation had little effect on plasticity (Radersma *et al*., 2020).

Finally, we expected SLA, root-to-shoot ratio and stomatal density to show no plasticity to combined heat and drought because plastic responses to heat and drought alone are in opposite directions that cancel out (Vile *et al*., 2012; Zhang and Sonnewald, 2017). Our results show plasticity in each of these traits; for example, stomatal density was unexpectedly plastic in both populations (Fig. 1D). Our stomatal density values are two to five times greater than the stomatal density reported in other chamber studies using *A. thaliana* populations from the native range (Vile *et al*., 2012; Lorts and Lasky, 2020) but about 33 % less than transgenic genotypes developed to increase stomatal density (Tanaka *et al*., 2013). Vile *et al*. (2012) found stomatal density was not plastic between treatments similar to ours though it plastically decreased in heat and increased in drought compared to the cool, well-watered treatment. We observed plasticity to increase stomatal density in the Future treatment relative to the Current treatment and greater stomatal density in the Swedish population than in the Italian population. Sweden and Italy may have differentiation for stomatal density from evolving in climates with different drought regimes. The contrast between our results and Vile *et al*. (2012) in stomatal density magnitude and plasticity even in the same species and similar chamber environments indicates that an underlying trait more directly involved in photosynthesis, such as stomatal conductance, may be more informative about drought response.

Further work should characterize if the observed plasticity is adaptive in both populations; our chamber study was limited in simulating field environments, and the sample sizes were too small to robustly test for adaptive plasticity (maximum sample size = 27 individuals but only 12 genotypes in the Swedish population). Given the genetic differentiation for drought strategy between the populations, the conserved plasticity may be adaptive in one population but neutral or even maladaptive in the other. For example, decreased SLA in heat and drought is expected of drought avoidant plants. Decreasing SLA could be adaptive plasticity for the Swedish population but maladaptive for the Italian population. Understanding if plasticity in the Swedish population is adaptive will help predictions of population survival in future climates.

### Local adaptation of the Swedish population is apparent in seeds per fruit but not fruit number

The total fitness of the Swedish population was not much higher than that of the Italian population in our Current treatment, probably because local adaptation in the field is driven by cold tolerance (Oakley *et al*., 2018, 2023; Lee *et al*., 2024), and our winter temperatures were well above freezing. The Swedish population did produce more seeds per fruit in the Current treatment, however, consistent with local adaptation. In the field, there was no difference in total fitness measured by fruits per seedling planted between the Swedish and Italian population at the Swedish field site in only one of eight years (2010; Ågren and Schemske, 2012; Ågren *et al*., 2013; Oakley *et al*., 2023), and if total fitness is measured by seeds per seedling planted, the Swedish population outperforms the Italian population in 2010 too (Ellis *et al*., 2021). Cold tolerance is also an important aspect of local adaptation in *A. thaliana* across the Eurasian range (Monroe *et al*., 2016). However, climate change is increasing winter temperatures (IPCC, 2021), so studies with higher winter air temperatures like ours may help predict the impact of climate change on local adaptation and the efficacy of conservation efforts such as assisted gene flow (reviewed in Anderson, 2023).

## CONCLUSIONS

We identified genetic differentiation between locally adapted populations of *A. thaliana* from Sweden and Italy that show Sweden avoids drought and Italy escapes drought. Despite this, both populations were plastic in the direction expected of drought avoidance when grown in a hotter and drier Swedish climate. Whether this evolutionarily conserved plasticity is adaptative remains unknown. Our hot and dry treatment greatly decreased the fitness of both populations, raising concerns about population persistence under abiotic stress.

## Supplementary Material

mcag153_Supplementary_Data

## SUPPLEMENTARY DATA

Supplementary data are available at *Annals of Botany* online and consist of the following. Table S1: Chamber conditions for the 2021 Experiment. Table S2: Chamber conditions for the 2022 Experiment. Table S3: ANOVA results for leaf fresh mass, leaf rehydrated mass, leaf dried mass, leaf area, leaf dry matter content, rosette mass, reproductive mass, leaf number, reproductive-to-rosette biomass allocation and seed weight. Table S4: ANOVA results for biomass traits from the 2022 Experiment after excluding the Roda-11 outlier. Figure S1: Reaction norms for leaf dry matter content and components of SLA and relative water content. Figure S2: Genotypic-level plasticity to illustrate within-population variation in plasticity. Figure S3: Reaction norms for biomass, leaf number and seed weight. All raw data and code are available through Zenodo at doi: 10.5281/zenodo.20400527.

## References

[mcag153-B1] Abbott RJ, Gomes MF. 1989. Population genetic structure and outcrossing rate of *Arabidopsis thaliana* (L) Heynh. Heredity 62: 411–418. doi: 10.1038/hdy.1989.56

[mcag153-B2] Ågren J, Oakley CG, Lundemo S, Schemske DW. 2017. Adaptive divergence in flowering time among natural populations of *Arabidopsis thaliana*: estimates of selection and QTL mapping. Evolution 71: 550–564. doi: 10.1111/evo.13126 27859214

[mcag153-B3] Ågren J, Oakley CG, Mckay JK, Lovell JT, Schemske DW. 2013. Genetic mapping of adaptation reveals fitness tradeoffs in *Arabidopsis thaliana*. Proceedings of the National Academy of Sciences of the United States of America 110: 21077–21082. doi: 10.1073/pnas.1316773110 PMC3876199 24324156

[mcag153-B4] Ågren J, Schemske DW. 2012. Reciprocal transplants demonstrate strong adaptive differentiation of the model organism *Arabidopsis thaliana* in its native range. New Phytologist 194: 1112–1122. doi: 10.1111/j.1469-8137.2012.04112.x 22432639

[mcag153-B5] Anderson JT . 2016. Plant fitness in a rapidly changing world. New Phytologist 210: 81–87. doi: 10.1111/nph.13693 26445400

[mcag153-B6] Anderson JT . 2023. The consequences of winter climate change for plant performance. American Journal of Botany 110: e16252. doi: 10.1002/ajb2.16252 37882251

[mcag153-B7] Bates D, Mächler M, Bolker B, Walker S. 2015. Fitting linear mixed-effects models using lme4. Journal of Statistical Software 67: 1–48. doi: 10.18637/JSS.V067.I01

[mcag153-B8] Bell DL, Galloway LF. 2008. Population differentiation for plasticity to light in an annual herb: adaptation and cost. American Journal of Botany 95: 59–65. doi: 10.3732/ajb.95.1.59 21632315

[mcag153-B9] Blum A . 2014. Genomics for drought resistance—getting down to earth. Functional Plant Biology: FPB 41: 1191–1198. doi: 10.1071/FP14018 32481068

[mcag153-B10] Bradshaw AD . 1965. Evolutionary significance of phenotypic plasticity in plants. Advances in Genetics 13: 115–155. doi: 10.1016/S0065-2660(08)60048-6

[mcag153-B11] Clauw P, Coppens F, De Beuf K, et al 2015. Leaf responses to mild drought stress in natural variants of Arabidopsis. Plant Physiology 167: 800–816. doi: 10.1104/pp.114.254284 PMC4348775 25604532

[mcag153-B12] Cohu CM, Muller O, Stewart JJ, Demmig-Adams B, Adams WW. 2013. Association between minor loading vein architecture and light- and CO_2_-saturated rates of photosynthetic oxygen evolution among *Arabidopsis thaliana* ecotypes from different latitudes. Frontiers in Plant Science 4: 264. doi: 10.3389/fpls.2013.00264 PMC3724126 23898338

[mcag153-B13] de Ollas C, Segarra C, Blázquez MA, Agustí J, Gómez-Cadenas A. 2023. Plant size directly correlates with water use efficiency in Arabidopsis. Plant, Cell & Environment 46: 2711–2725. doi: 10.1111/pce.14663 37427824

[mcag153-B14] Des Marais DL, Hernandez KM, Juenger TE. 2013. Genotype-by-environment interaction and plasticity: exploring genomic responses of plants to the abiotic environment. Annual Review of Ecology, Evolution, and Systematics 44: 5–29. doi: 10.1146/annurev-ecolsys-110512-135806

[mcag153-B15] Des Marais DL, Mckay JK, Richards JH, Sen S, Wayne T, Juenger TE. 2012. Physiological genomics of response to soil drying in diverse Arabidopsis accessions. Plant Cell 24: 893–914. doi: 10.1105/tpc.112.096180 PMC3336118 22408074

[mcag153-B16] Diamond SE, Martin RA. 2021. Buying time: plasticity and population persistence. In: Pfennig DW. ed. Phenotypic plasticity & evolution. Boca Raton: CRC Press, 139–160.

[mcag153-B17] Dittberner H, Korte A, Mettler-Altmann T, Weber APM, Monroe G, de Meaux J. 2018. Natural variation in stomata size contributes to the local adaptation of water-use efficiency in *Arabidopsis thaliana*. Molecular Ecology 27: 4052–4065. doi: 10.1111/mec.14838 PMC7611081 30118161

[mcag153-B18] Dittmar EL, Oakley CG, Ågren J, Schemske DW. 2014. Flowering time QTL in natural populations of *Arabidopsis thaliana* and implications for their adaptive value. Molecular Ecology 23: 4291–4303. doi: 10.1111/mec.12857 25039363

[mcag153-B19] Dudley SA, Schmitt J. 1996. Testing the adaptive plasticity hypothesis: density-dependent selection on manipulated stem length in *Impatiens capensis*. The American Naturalist 147: 445–465. doi: 10.1086/285860

[mcag153-B20] Durán P, Ellis TJ, Thiergart T, Ågren J, Hacquard S. 2022. Climate drives rhizosphere microbiome variation and divergent selection between geographically distant *Arabidopsis* populations. New Phytologist 236: 608–621. doi: 10.1111/nph.18357 35794837

[mcag153-B21] Ellis TJ, Ågren J. 2024. Adaptation to soil type contributes little to local adaptation in an Italian and a Swedish ecotype of *Arabidopsis thaliana* on contrasting soils. Biology Letters 20: 20240236. doi: 10.1098/rsbl.2024.0236 PMC11387056 39255844

[mcag153-B22] Ellis TJ, Postma FM, Oakley CG, Ågren J. 2021. Life-history trade-offs and the genetic basis of fitness in *Arabidopsis thaliana*. Molecular Ecology 30: 2846–2858. doi: 10.1111/mec.15941 33938082

[mcag153-B23] Estarague A, Vasseur F, Sartori K, et al 2022. Into the range: a latitudinal gradient or a center-margins differentiation of ecological strategies in *Arabidopsis thaliana*? Annals of Botany 129: 343–356. doi: 10.1093/aob/mcab149 PMC8835660 34918027

[mcag153-B24] Franks SJ, Weber JJ, Aitken SN. 2014. Evolutionary and plastic responses to climate change in terrestrial plant populations. Evolutionary Applications 7: 123–139. doi: 10.1111/eva.12112 PMC3894902 24454552

[mcag153-B25] Gao G, Tester MA, Julkowska MM. 2020. The use of high-throughput phenotyping for assessment of heat stress-induced changes in Arabidopsis. Plant Phenomics 2020: 3723916. doi: 10.34133/2020/3723916 PMC7706305 33313552

[mcag153-B26] Ghalambor CK, McKay JK, Carroll SP, Reznick DN. 2007. Adaptive versus non-adaptive phenotypic plasticity and the potential for contemporary adaptation in new environments. Functional Ecology 21: 394–407. doi: 10.1111/j.1365-2435.2007.01283.x

[mcag153-B27] Gianoli E, González-Teuber M. 2005. Environmental heterogeneity and population differentiation in plasticity to drought in *Convolvulus chilensis* (Convolvulaceae). Evolutionary Ecology 19: 603–613. doi: 10.1007/s10682-005-2220-5

[mcag153-B28] Granier C, Aguirrezabal L, Chenu K, et al 2006. PHENOPSIS, an automated platform for reproducible phenotyping of plant responses to soil water deficit in *Arabidopsis thaliana* permitted the identification of an accession with low sensitivity to soil water deficit. New Phytologist 169: 623–635. doi: 10.1111/j.1469-8137.2005.01609.x 16411964

[mcag153-B29] Grillo MA, Li C, Hammond M, Wang L, Schemske DW. 2013. Genetic architecture of flowering time differentiation between locally adapted populations of *Arabidopsis thaliana*. New Phytologist 197: 1321–1331. doi: 10.1111/nph.12109 23311994

[mcag153-B30] Hamann E, Denney D, Day S, et al 2021. Review: plant eco-evolutionary responses to climate change: emerging directions. Plant Science 304: 110737. doi: 10.1016/j.plantsci.2020.110737 33568289

[mcag153-B31] Handelsman CA, Broder ED, Dalton CM, et al 2013. Predator-induced phenotypic plasticity in metabolism and rate of growth: rapid adaptation to a novel environment. Integrative and Comparative Biology 53: 975–988. doi: 10.1093/icb/ict057 23784701

[mcag153-B32] Heblack J, Schepers JR, Willi Y. 2024. Evolutionary potential under heat and drought stress at the southern range edge of North American *Arabidopsis lyrata*. Journal of Evolutionary Biology 37: 555–565. doi: 10.1093/jeb/voae045 38596851

[mcag153-B33] IPCC . 2021. Climate Change 2021: The Physical Science Basis. Contribution of Working Group I to the Sixth Assessment Report of the Intergovernmental Panel on Climate Change. Cambridge, United Kingdom and New York, NY, USA: Cambridge University Press.

[mcag153-B34] Jiang Z, van Zanten M, Sasidharan R. 2025. Mechanisms of plant acclimation to multiple abiotic stresses. Communications Biology 8: 655. doi: 10.1038/s42003-025-08077-w PMC12019247 40269242

[mcag153-B35] Juenger TE, Mckay JK, Hausmann N, et al 2005. Identification and characterization of QTL underlying wholeplant physiology in *Arabidopsis thaliana*: δ^13^C, stomatal conductance and transpiration efficiency. Plant, Cell & Environment 28: 697–708. doi: 10.1111/j.1365-3040.2004.01313.x

[mcag153-B36] Kawecki TJ, Ebert D. 2004. Conceptual issues in local adaptation. Ecology Letters 7: 1225–1241. doi: 10.1111/j.1461-0248.2004.00684.x

[mcag153-B37] Kenney AM, Mckay JK, Richards JH, Juenger TE. 2014. Direct and indirect selection on flowering time, water-use efficiency (WUE, δ^13^C), and WUE plasticity to drought in *Arabidopsis thaliana*. Ecology and Evolution 4: 4505–4521. doi: 10.1002/ece3.1270 PMC4264900 25512847

[mcag153-B38] Kong RS, Henry HAL. 2016. Prior exposure to freezing stress enhances the survival and recovery of *Poa pratensis* exposed to severe drought. American Journal of Botany 103: 1890–1896. doi: 10.3732/ajb.1600176 27803002

[mcag153-B39] Kooyers NJ . 2015. The evolution of drought escape and avoidance in natural herbaceous populations. Plant Science 234: 155–162. doi: 10.1016/j.plantsci.2015.02.012 25804818

[mcag153-B40] Kuznetsova A, Brockhoff PB, Christensen RHB. 2017. lmerTest package: tests in linear mixed effects models. Journal of Statistical Software 82: 1–26. doi: 10.18637/JSS.V082.I13

[mcag153-B41] Laitinen RAE, Nikoloski Z. 2019. Genetic basis of plasticity in plants. Journal of Experimental Botany 70: 739–745. doi: 10.1093/jxb/ery404 30445526

[mcag153-B42] Lee G, Sanderson BJ, Ellis TJ, et al 2024. A large-effect fitness trade-off across environments is explained by a single mutation affecting cold acclimation. Proceedings of the National Academy of Sciences of the United States of America 121: e2317461121. doi: 10.1073/pnas.2317461121 PMC10861903 38289961

[mcag153-B43] Lenth RV . 2024. Emmeans: estimated marginal Means, aka Least Squares Means. doi: 10.32614/CRAN.package.emmeans.

[mcag153-B44] Levitt J . 1980. Water, radiation, salt, and other stresses. New York: Academic Press.

[mcag153-B45] Lorts CM, Lasky JR. 2020. Competition × drought interactions change phenotypic plasticity and the direction of selection on Arabidopsis traits. New Phytologist 227: 1060–1072. doi: 10.1111/nph.16593 32267968

[mcag153-B46] Lovell JT, Juenger TE, Michaels SD, et al 2013. Pleiotropy of FRIGIDA enhances the potential for multivariate adaptation. Proceedings of the Royal Society of London: Series B, Biological Sciences 280: 20131043. doi: 10.1098/rspb.2013.1043 PMC3774242 23698015

[mcag153-B47] Ludlow MM . 1989. Strategies of response to water stress. In: Kreeb KH, Richter H, Hinckley TM. eds. Structural and functional responses to environmental stresses: water shortage. The Hague: SPB Academic Publ, 269–281.

[mcag153-B48] Mckay JK, Richards JH, Mitchell-Olds T. 2003. Genetics of drought adaptation in *Arabidopsis thaliana*: I. Pleiotropy contributes to genetic correlations among ecological traits. Molecular Ecology 12: 1137–1151. doi: 10.1046/j.1365-294X.2003.01833.x 12694278

[mcag153-B49] Michaels SD, Amasino RM. 2000. Memories of winter: vernalization and the competence to flower. Plant, Cell & Environment 23: 1145–1153. doi: 10.1046/j.1365-3040.2000.00643.x

[mcag153-B50] Mojica JP, Mullen J, Lovell JT, et al 2016. Genetics of water use physiology in locally adapted *Arabidopsis thaliana*. Plant Science 251: 12–22. doi: 10.1016/j.plantsci.2016.03.015 27593459

[mcag153-B51] Monroe JG, McGovern C, Lasky JR, Grogan K, Beck J, McKay JK. 2016. Adaptation to warmer climates by parallel functional evolution of CBF genes in *Arabidopsis thaliana*. Molecular Ecology 25: 3632–3644. doi: 10.1111/mec.13711 27247130

[mcag153-B52] Morris MRJ . 2014. Plasticity-mediated persistence in new and changing environments. International Journal of Evolutionary Biology 2014: 416497. doi: 10.1155/2014/416497 PMC4216699 25386380

[mcag153-B53] Murren CJ, Maclean HJ, Diamond SE, et al 2014. Evolutionary change in continuous reaction norms. The American Naturalist 183: 453–467. doi: 10.1086/675302 24642491

[mcag153-B54] Oakley CG, Ågren J, Atchison RA, Schemske DW. 2014. QTL mapping of freezing tolerance: links to fitness and adaptive trade-offs. Molecular Ecology 23: 4304–4315. doi: 10.1111/mec.12862 25039860

[mcag153-B55] Oakley CG, Ågren J, Schemske DW. 2015. Heterosis and outbreeding depression in crosses between natural populations of *Arabidopsis thaliana*. Heredity 115: 73–82. doi: 10.1038/hdy.2015.18 PMC4815493 26059971

[mcag153-B56] Oakley CG, Savage L, Lotz S, et al 2018. Genetic basis of photosynthetic responses to cold in two locally adapted populations of *Arabidopsis thaliana*. Journal of Experimental Botany 69: 699–709. doi: 10.1093/jxb/erx437 PMC5853396 29300935

[mcag153-B57] Oakley CG, Schemske DW, McKay JK, Ågren J. 2023. Ecological genetics of local adaptation in Arabidopsis: an 8-year field experiment. Molecular Ecology 32: 4570–4583. doi: 10.1111/mec.17045 37317048

[mcag153-B58] Pérez-Harguindeguy N, Díaz S, Garnier E, et al 2013. New handbook for standardised measurement of plant functional traits worldwide. Australian Journal of Botany 61: 167. doi: 10.1071/BT12225

[mcag153-B59] Pfennig DW . 2021. Key questions about phenotypic plasticity. In: Pfennig DW. ed. Phenotypic plasticity & evolution. Boca Raton: CRC Press, 55–88.

[mcag153-B60] Platt A, Horton M, Huang YS, et al 2010. The scale of population structure in *Arabidopsis thaliana*. PLoS Genetics 6: 1000843. doi: 10.1371/journal.pgen.1000843 PMC2820523 20169178

[mcag153-B61] Postma FM, Ågren J. 2016. Early life stages contribute strongly to local adaptation in *Arabidopsis thaliana*. Proceedings of the National Academy of Sciences of the United States of America 113: 7590–7595. doi: 10.1073/pnas.1606303113 PMC4941467 27330113

[mcag153-B62] Postma FM, Ågren J. 2018. Among-year variation in selection during early life stages and the genetic basis of fitness in *Arabidopsis thaliana*. Molecular Ecology 27: 2498–2511. doi: 10.1111/mec.14697 29676059

[mcag153-B63] Postma FM, Lundemo S, Ågren J. 2016. Seed dormancy cycling and mortality differ between two locally adapted populations of *Arabidopsis thaliana*. Annals of Botany 117: 249–256. doi: 10.1093/aob/mcv171 PMC4724045 26637384

[mcag153-B64] Price N, Moyers BT, Lopez L, et al 2018. Combining population genomics and fitness QTLs to identify the genetics of local adaptation in *Arabidopsis thaliana*. Proceedings of the National Academy of Sciences of the United States of America 115: 5028–5033. doi: 10.1073/PNAS.1719998115 PMC5948977 29686078

[mcag153-B65] Price TD, Qvarnström A, Irwin DE. 2003. The role of phenotypic plasticity in driving genetic evolution. Proceedings of the Royal Society of London: Series B, Biological Sciences 270: 1433–1440. doi: 10.1098/rspb.2003.2372 PMC1691402 12965006

[mcag153-B66] Price TD, Yeh PJ, Harr B. 2008. Phenotypic plasticity and the evolution of a socially selected trait following colonization of a novel environment. The American Naturalist 172: S49–S62. doi: 10.1086/588257 18554144

[mcag153-B67] Radersma R, Noble DWA, Uller T. 2020. Plasticity leaves a phenotypic signature during local adaptation. Evolution Letters 4: 360–370. doi: 10.1002/evl3.185 PMC7403707 32774884

[mcag153-B68] R Core Team . 2024. R: a language and environment for statistical computing. Vienna: R Foundation for Statistical Computing,. https://www.R-project.org/

[mcag153-B69] Rizhsky L, Liang H, Shuman J, Shulaev V, Davletova S, Mittler R. 2004. When defense pathways collide. The response of Arabidopsis to a combination of drought and heat stress. Plant Physiology 134: 1683–1696. doi: 10.1104/pp.103.033431 PMC419842 15047901

[mcag153-B70] Royer AM, Kremer C, George K, Pérez SG, Schemske DW, Conner JK. 2016. Incomplete loss of a conserved trait: function, latitudinal cline, and genetic constraints. Evolution 70: 2853–2864. doi: 10.1111/evo.13096

[mcag153-B71] Sanderson BJ, Park S, Jameel MI, et al 2020. Genetic and physiological mechanisms of freezing tolerance in locally adapted populations of a winter annual. American Journal of Botany 107: 250–261. doi: 10.1002/ajb2.1385 PMC7065183 31762012

[mcag153-B72] Scheiner SM . 1998. The genetics of phenotypic plasticity. VII. Evolution in a spatially-structured environment. Journal of Evolutionary Biology 11: 303–320. doi: 10.1007/s000360050090

[mcag153-B73] Schepers JR, Heblack J, Willi Y. 2024. Negative interaction effect of heat and drought stress at the warm end of species distribution. Oecologia 204: 173–185. doi: 10.1007/s00442-023-05497-5 PMC10830594 38253704

[mcag153-B74] Schneider CA, Rasband WS, Eliceiri KW. 2012. NIH Image to ImageJ: 25 years of image analysis. Nature Methods 9: 671–675. doi: 10.1038/nmeth.2089 PMC5554542 22930834

[mcag153-B75] Secomandi E, De Gregorio MA, Castro-Cegrí A, Lucini L. 2025. Biochemical, photosynthetic and metabolomics insights of single and combined effects of salinity, heat, cold and drought in Arabidopsis. Physiologia Plantarum 177: e70062. doi: 10.1111/ppl.70062 PMC11739553 39821073

[mcag153-B76] Stewart JJ, Adams WW, Cohu CM, Polutchko SK, Lombardi EM, Demmig-Adams B. 2015. Differences in light-harvesting, acclimation to growth-light environment, and leaf structural development between Swedish and Italian ecotypes of *Arabidopsis thaliana*. Planta 242: 1277–1290. doi: 10.1007/s00425-015-2368-3 26189001

[mcag153-B77] Stewart JJ, Demmig-Adams B, Cohu CM, Wenzl CA, Muller O, Adams III WW. 2016. Growth temperature impact on leaf form and function in *Arabidopsis thaliana* ecotypes from northern and southern Europe. Plant, Cell & Environment 39: 1549–1558. doi: 10.1111/pce.12720. 26832121

[mcag153-B78] Sultan SE, Spencer HG. 2002. Metapopulation structure facros plasticity over local adaptation. The American Naturalist 160: 271–283. doi: 10.1086/341015 18707492

[mcag153-B79] Suprasanna P, Nikalje GC, Rai AN. 2016. Osmolyte accumulation and implications in plant abiotic stress tolerance. In: Iqbal N, Nazar R, Khan NA. eds. Osmolytes and plants acclimation to changing environment: emerging omics technologies. New Delhi: Springer, 1–12.

[mcag153-B80] Suter L, Widmer A. 2013. Phenotypic effects of salt and heat stress over three generations in *Arabidopsis thaliana*. PLoS One 8: e80819. doi: 10.1371/journal.pone.0080819 PMC3828257 24244719

[mcag153-B81] Suzuki N, Rivero RM, Shulaev V, Blumwald E, Mittler R. 2014. Abiotic and biotic stress combinations. New Phytologist 203: 32–43. doi: 10.1111/nph.12797 24720847

[mcag153-B82] Tanaka Y, Sugano SS, Shimada T, Hara-Nishimura I. 2013. Enhancement of leaf photosynthetic capacity through increased stomatal density in Arabidopsis. New Phytologist 198: 757–764. doi: 10.1111/nph.12186 23432385

[mcag153-B83] VanBuren R, Nguyen A, Marks RA, et al 2024. Variability in drought gene expression datasets highlight the need for community standardization. *bioRxiv* 2024.02.04.578814. doi: 10.1101/2024.02.04.578814. PMC12854406 41401198

[mcag153-B84] Van Buskirk J . 2002. A comparative test of the adaptive plasticity hypothesis: relationships between habitat and phenotype in anuran larvae. The American Naturalist 160: 87–102. doi: 10.1086/340599 18707501

[mcag153-B85] Vasseur F, Pantin F, Vile D. 2011. Changes in light intensity reveal a major role for carbon balance in Arabidopsis responses to high temperature. Plant, Cell & Environment 34: 1563–1576. doi: 10.1111/j.1365-3040.2011.02353.x 21707647

[mcag153-B86] Verslues PE, Juenger TE. 2011. Drought, metabolites, and Arabidopsis natural variation: a promising combination for understanding adaptation to water-limited environments. Current Opinion in Plant Biology 14: 240–245. doi: 10.1016/j.pbi.2011.04.006 21561798

[mcag153-B87] Vile D, Pervent M, Belluau M, et al 2012. Arabidopsis growth under prolonged high temperature and water deficit: independent or interactive effects? Plant, Cell & Environment 35: 702–718. doi: 10.1111/j.1365-3040.2011.02445.x 21988660

[mcag153-B88] Wickham H . 2016. ggplot2: Elegant Graphics for Data Analysis. https://ggplot2.tidyverse.org

[mcag153-B89] Wickham H, Averick M, Bryan J, et al 2019. Welcome to the Tidyverse. Journal of Open Source Software 4: 1686. doi: 10.21105/joss.01686

[mcag153-B90] Yeh PJ, Price TD. 2004. Adaptive phenotypic plasticity and the successful colonization of a novel environment. The American Naturalist 164: 531–542. doi: 10.1086/423825 15459883

[mcag153-B91] Zacchello G, Vinyeta M, Ågren J. 2020. Strong stabilizing selection on timing of germination in a Mediterranean population of *Arabidopsis thaliana*. American Journal of Botany 107: 1518–1526. doi: 10.1002/ajb2.1549 PMC7756891 33058187

[mcag153-B92] Zandalinas SI, Mittler R. 2022. Plant responses to multifactorial stress combination. New Phytologist 234: 1161–1167. doi: 10.1111/nph.18087 35278228

[mcag153-B93] Zhang H, Sonnewald U. 2017. Differences and commonalities of plant responses to single and combined stresses. The Plant Journal 90: 839–855. doi: 10.1111/tpj.13557 28370754

