# Supplementary material for: Differentiation for drought strategy but conserved plasticity to heat and drought in locally adapted populations of Arabidopsis thaliana
Source: Ann Bot. 2026 May 27;138(4):993–1006. doi: 10.1093/aob/mcag153 (PMC13595241; doi:10.1093/aob/mcag153)
Supplement: Supplementary file 1 [file mcag153_supplementary_data.pdf]

Table S1. Temperatures (°C) and soil moisture (%) for treatments in the 2021 experiment with their corresponding dates from the Sweden field site. Temperatures are written as Day Temperature:Night Temperature. Day lengths are denoted as hours:minutes.

Week	Current		Future		Day Length	Simulated Dates
	Temp	Soil Moisture	Temp	Soil Moisture		
1	16:13	45	20:17	25	15:45	Aug 1 - 23
2	14:11	45	18:15	25	14:15	Aug 24 – Sept 15
3	8:6	45	12:10	25	12:00	Sept 16 – Oct 8
4	8:6	45	10:9	25	9:30	Oct 9 – 31
5	10:8	45	10:8	25	7:00	Nov 1 – Dec 5
6	10:8	45	10:8	25	5:00	Dec 6 – Jan 7
7	10:8	45	10:8	25	7:00	Jan 8 – Feb 11
8	10:8	45	10:8	25	10:00	Feb 12 – Mar 16
9	10:8	45	10:8	25	13:30	Mar 17 – Apr 20
10	8:6	45	10:7	10	15:45	Apr 21 – 27
11	8:6	45	12:7	10	16:30	Apr 28 – May 4
12	9:6	45	13:10	10	17:00	May 5 – 11
13	10:6	45	14:7	7.5	17:45	May 12 – 18
14	9:6	45	13:10	7.5	18:30	May 19 – 25
15	9:6	45	13:10	7.5	19:00	May 26 – Jun 1
16	12:9	45	16:13	7.5	19:30	June 2 – 8
17	10:9	45	14:13	7.5	19:45	June 9 – 15
18	15:11	45	19:15	7.5	20:00	June 16 – 22
19	14:11	45	18:15	7.5	20:00	June 23 – 29
20	18:12.5	45	22:16.5	7.5	19:45	June 30 – July 6
21	20:13.5	45	24:17.5	7.5	19:15	July 7 – 13
22	16:13	45	20:17	7.5	18:45	July 14 – 20
23	16:13	45	20:17	7.5	18:45	July 14 – 20
24	16:12	45	20:16	7.5	18:15	July 21 – 27
25	18:13	45	22:17	7.5	17:30	July 28 – Aug 3

Table S2. Temperatures (°C) and soil moisture (%) for treatments in the 2022 experiment with their corresponding dates from the Sweden field site if applicable. Temperatures are written as Day Temperature:Night Temperature. Day lengths are denoted as hours:minutes. Plants were well-watered when planted, and Future plants did not reach the target soil moisture levels until multiple weeks post planting, with some not reaching 7.5% until the spring simulation.

Week	Current		Future		Day Length	Simulated Dates
	Temp	Soil Moisture	Temp	Soil Moisture		
1	16:13	45	16:13	7.5	16:00	Fall
2	16:13	45	16:13	7.5	16:00	Fall
3	16:13	45	16:13	7.5	16:00	Fall
4	10:8	45	10:8	7.5	7:00	Nov 1 – Dec 5
5	10:8	45	10:8	7.5	5:00	Dec 6 – Jan 7
6	10:8	45	10:8	7.5	7:00	Jan 8 – Feb 11
7	10:8	45	10:8	7.5	10:00	Feb 12 – Mar 16
8	10:8	45	10:8	7.5	13:30	Mar 17 – Apr 20
9	10:6	45	14:10	7.5	17:00	May 5 – 11
10	10:6	45	14:10	7.5	17:45	May 12 – 18
11	10:6	45	14:10	7.5	18:30	May 19 – 25
12	10:6	45	14:10	7.5	19:00	May 26 – Jun 1
13	12:9	45	16:13	7.5	19:30	June 2 – 8
14	10:9	45	14:13	7.5	19:45	June 9 – 15
15	15:11	45	19:15	10	20:00	June 16 – 22
16	14:11	45	18:15	10	20:00	June 23 – 29
17	18:12.5	45	22:16.5	10	19:45	June 30 – July 6
18	20:13.5	45	24:17.5	10	19:15	July 7 – 13

Table S3. Anova results for all traits in supplemental. Columns denote the trait used as the response variable in the model, the predictor model terms, and then for the 2021 and 2022 experiments the sum of squares, the denominator degrees of freedom, *F* value, and *p* value. Numerator degrees of freedom is always 1 for treatment, population, and their interaction; it is always 2 for block. All single leaf mass and biomass values were measured in grams (g). Symbols illustrate *p* value: † < 0.1; * <0.05; ** < 0.01; *** < 0.001.

Experiment		2021				2022			
Trait	Term	SumSq	DenDF	*F*	*p*	SumSq	DenDF	*F*	*p*
Log Fresh Mass	Treatment	4.317	62.230	336.925	8.407E-27***	0.222	106.655	17.172	6.863E-05 ***
	Population	0.009	16.438	0.735	0.403	0.126	12.353	9.759	0.009 **
	Interaction	0.117	62.230	9.139	0.004 **	0.051	106.787	3.959	0.049 *
	Block	Not applicable				0.003	106.825	0.115	0.892
Log Rehydrated Mass	Treatment	3.889	62.412	302.877	1.246E-25***	0.114	105.702	8.846	0.004 **
	Population	0.011	16.515	0.841	0.372	0.097	12.403	7.471	0.018 *
	Interaction	0.098	62.412	7.634	0.008 **	0.080	105.863	6.170	0.015 *
	Block	Not applicable				0.005	105.903	0.185	0.831
Log Dried Mass	Treatment	1.710	62.401	129.558	7.219E-17***	4.221E-06	107.128	1.054	0.307
	Population	0.030	16.304	2.282	0.150	1.024E-04	12.734	25.571	2.343E-04 ***
	Interaction	0.021	62.401	1.577	0.214	8.301E-06	107.266	2.074	0.153
	Block	Not applicable				8.432E-06	107.311	1.053	0.352
Log Leaf Area	Treatment	3.387	60.599	342.253	1.307E-26***	4.555	106.079	14.583	2.262E-04 ***
	Population	0.004	15.174	0.414	0.529	7.468E-05	11.621	2.391E-04	0.988
	Interaction	0.167	60.599	16.867	1.221E-04***	1.698	106.241	5.435	0.022 **
	Block	Not applicable				0.287	106.307	0.460	0.633

Experiment		2021				2022			
Trait	Term	SumSq	DenDF	*F*	*p*	SumSq	DenDF	*F*	*p*
Log Leaf Dry Matter Content	Treatment	0.444	63.844	258.377	3.997E-24***	0.246	119.000	44.563	8.267E-10 ***
	Population	0.006	15.805	3.349	0.086 †	0.246	119.000	44.581	8.211E-10 ***
	Interaction	0.029	63.844	16.678	1.258E-04***	0.018	119.000	3.313	0.071 †
	Block	Not applicable				0.036	119.000	3.241	0.043 *
Log Dried Rosette Mass	Treatment	1.250	59.024	70.410	1.193E-11***	Not measured			
	Population	0.059	14.375	3.300	0.090†				
	Interaction	0.024	59.024	1.347	0.250				
Log Dry Repro Mass	Treatment	11.162	66.020	249.115	4.285E-24***	Not measured			
	Population	0.009	21.705	0.205	0.655				
	Interaction	0.007	66.020	0.165	0.686				
Leaf Number at bolting	Treatment	Not measured				0.001	107.502	0.098	0.754
	Population					0.244	13.128	41.834	2.010E-05 ***
	Interaction					0.007	107.635	1.186	0.279
	Block					0.007	107.677	0.571	0.567

Experiment		2021				2022			
Trait	Term	SumSq	DenDF	*F*	*p*	SumSq	DenDF	*F*	*p*
Leaf Number at harvest	Treatment	1505.028	61.065	125.530	1.899E-16***	Not measured			
	Population	183.535	15.359	15.308	0.001***				
	Interaction	19.502	61.065	1.627	0.207				
Log Repro:Rosette	Treatment	4.645	61.668	108.165	3.417E-15***	Not measured			
	Population	0.237	18.173	5.524	0.030*				
	Interaction	0.052	61.668	1.202	0.277				
Average Seed Weight	Treatment	1.230E-05	57.379	0.855	0.359	Not measured			
	Population	1.138E-04	12.151	7.910	0.016 **				
	Interaction	2.969E-06	57.379	0.206	0.651				

Table S4. Anova results for biomass traits from the 2022 Experiment with outlier genotype Roda-11 removed from analyses. Columns denoted as in Table S3. Symbols illustrate *p* value: † < 0.1; * <0.05; ** < 0.01; *** < 0.001. Bolded *p*-values are those that changed significance level when the outlier was removed.

Experiment		2022			
Trait	Term	SumSq	DenDF	*F*	*p*
Root:shoot Ratio	Treatment	0.002	100.759	0.739	0.392
	Population	0.030	12.538	12.781	0.004 **
	Interaction	0.052	100.857	22.351	**7.387E-06** ***[a]
	Block	0.006	100.912	1.311	0.274
Total Biomass (g)	Treatment	0.042	101.228	3.277	**0.073** †[b]
	Population	0.488	12.704	38.404	3.581E-05 ***
	Interaction	0.015	101.351	1.209	0.274
	Block	0.002	101.458	0.090	0.914
Root Biomass (g)	Treatment	8.220E-04	101.184	5.881	**0.017** *[c]
	Population	0.003	12.658	23.776	3.27E-04 ***
	Interaction	1.649E-5	101.201	0.118	0.731
	Block	1.598E-04	101.295	0.571	0.566
Shoot Biomass (g)	Treatment	0.035	100.358	2.568	0.112
	Population	0.368	12.175	26.972	2.140E-04 ***
	Interaction	0.070	100.445	5.125	0.026 *
	Block	0.002	100.491	0.066	0.936

[a] p-value decreased from 0.003
[b] p-value decreased from 0.109
[c] p-value decreased from 0.085

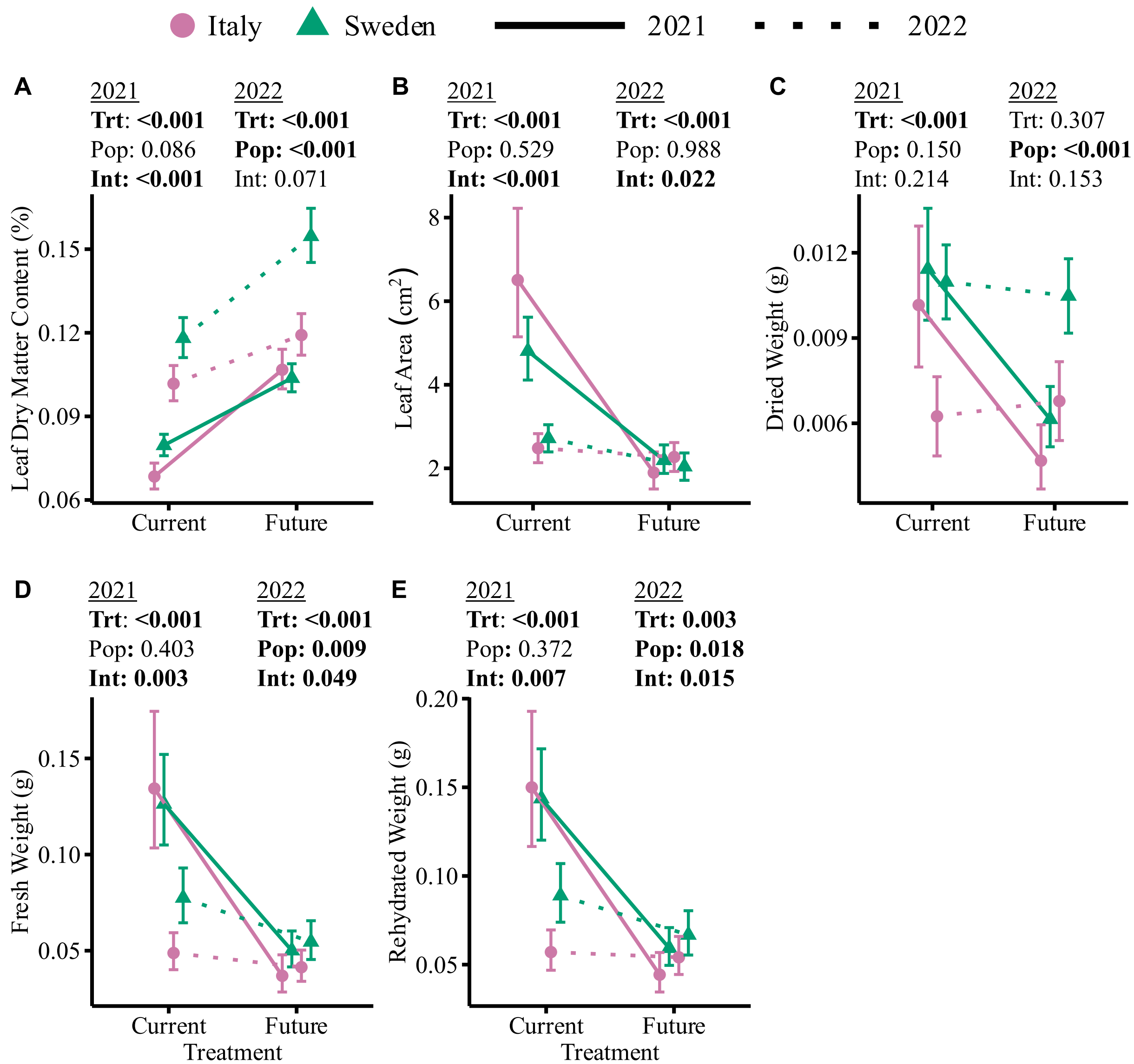

Figure S1. Reaction norms for leaf dry matter content and components of specific leaf area and relative water content measured at bolting (2022) or flowering (2021). Each panel displays estimated marginal means for Sweden (triangles) and Italy (circles) with 95% confidence intervals. Lines connecting means illustrate plasticity; the 2021 experiment is connected with solid lines and the 2022 experiment with dotted lines. Statistics above each panel show *p*-values for Anova fixed effects. Leaf dry matter content, fresh weight, and rehydrated weight were log transformed for statistical analysis in both experiments. Leaf area and dry weight were log transformed in 2021 only.

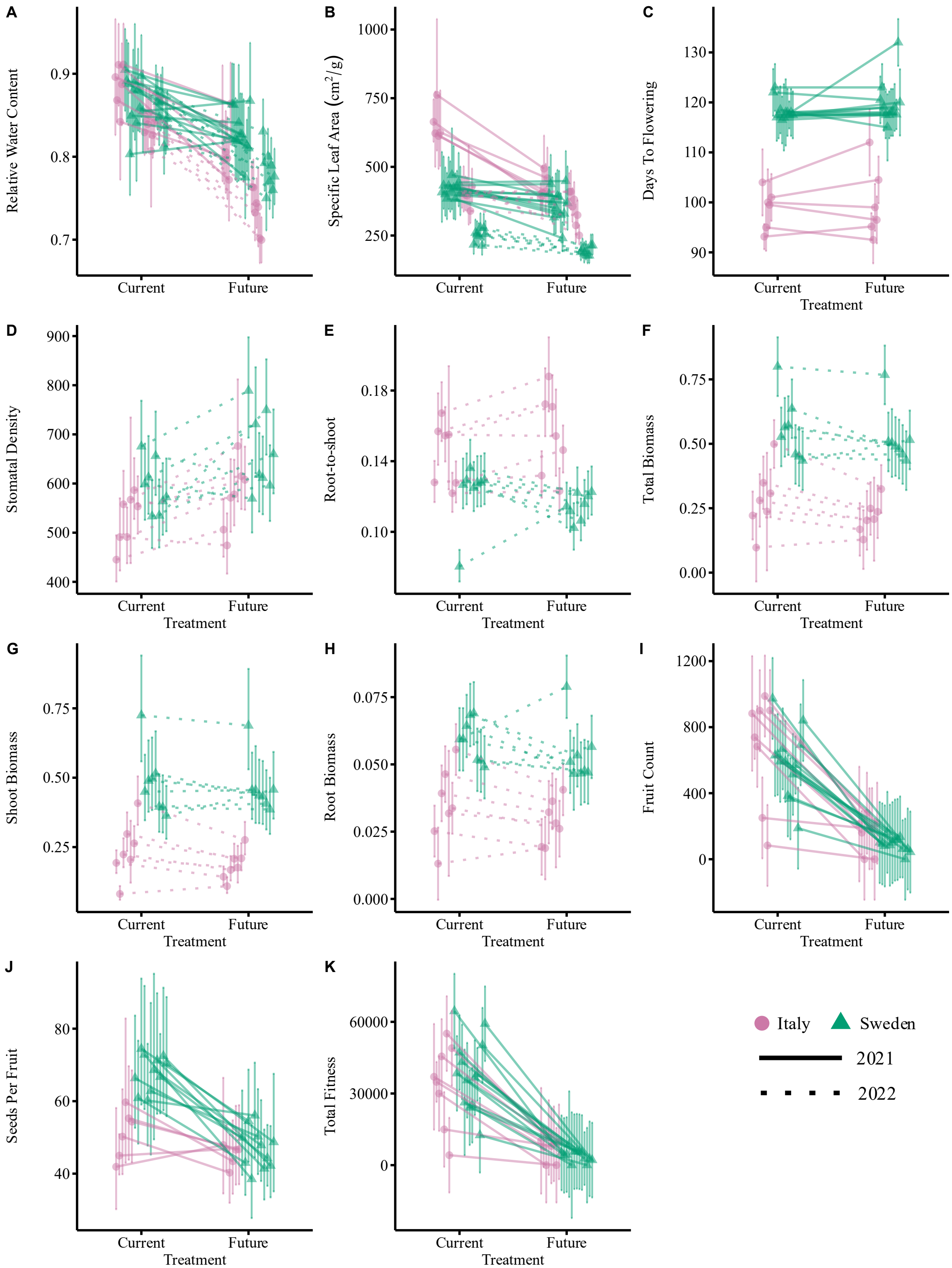

Figure S2. Genotypic-level plasticity shows within-population variation in plasticity to heat and drought. Each panel displays estimated marginal means for Swedish genotypes (triangles) and Italian genotypes (circles) with 95% confidence intervals. Lines connecting means illustrate plasticity; there is one line per genotype. The 2021 experiment is connected with solid lines and the 2022 experiment with dotted lines. See Table S4 for outlier removal information.

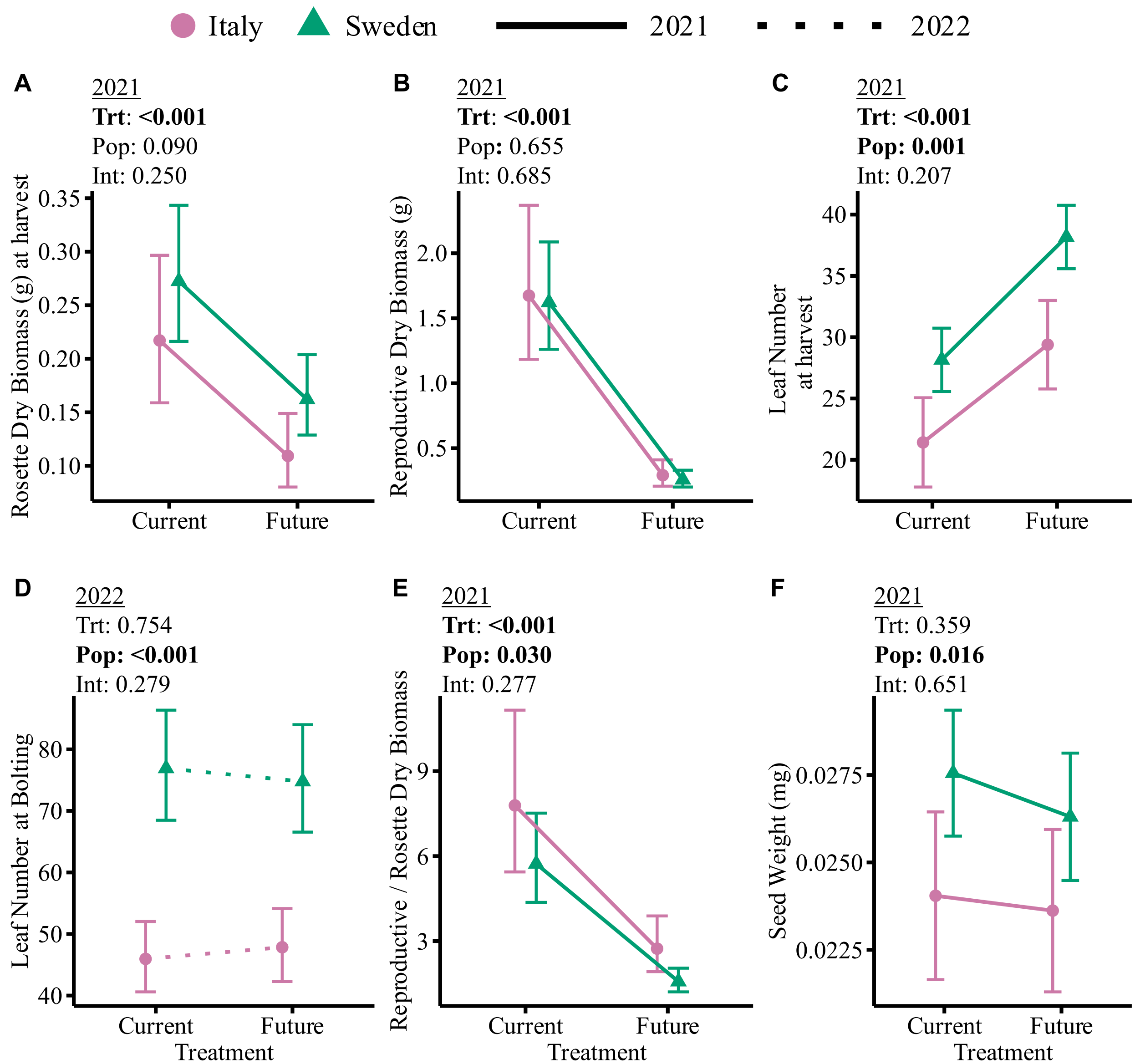

Figure S3. Reaction norms for biomass, leaf number, and seed weight. Each panel displays estimated marginal means for Sweden (triangles) and Italy (circles) with 95% confidence intervals. Lines connecting means illustrate plasticity; the 2021 experiment is connected with solid lines and the 2022 experiment with dotted lines. Statistics above each panel show *p*-values for Anova fixed effects. Rosette biomass, reproductive biomass and the ratio of reproductive:rosette biomass were log transformed for analysis. Note the difference in the y axis between panel C and D; rosettes in the 2022 experiment developed axillary meristem growth during the vegetative phase which greatly increased leaf number.
